# Some linguistic neutrosophic Hamy mean operators and their application to multi-attribute group decision making

**DOI:** 10.1371/journal.pone.0193027

**Published:** 2018-03-07

**Authors:** Peide Liu, Xinli You

**Affiliations:** School of Management Science and Engineering, Shandong University of Finance and Economics, Jinan Shandong, China; Zhongnan University of Economics and Law, CHINA

## Abstract

Linguistic neutrosophic numbers (LNNs) can easily describe the incomplete and indeterminate information by the truth, indeterminacy, and falsity linguistic variables (LVs), and the Hamy mean (HM) operator is a good tool to deal with multiple attribute group decision making (MAGDM) problems because it can capture the interrelationship among the multi-input arguments. Motivated by these ideas, we develop linguistic neutrosophic HM (LNHM) operator and weighted linguistic neutrosophic HM (WLNHM) operator. Some desirable properties and special cases of two operators are discussed in detail. Furthermore, considering the situation in which the decision makers (DMs) can’t give the suitable weight of each attribute directly from various reasons, we propose the concept of entropy for linguistic neutrosophic set (LNS) to obtain the attribute weight vector objectively, and then the method for MAGDM problems with LNNs is proposed, and some examples are used to illustrate the effectiveness and superiority of the proposed method by comparing with the existing methods.

## 1. Introduction

Nowadays, the multi-attribute decision-making (MADM) or MAGDM is widely existed in various fields [1–5], and to obtain accurate evaluation information is one of premises for DMs to make rational and feasible decision. However, in real-world situation, there are a variety of limitations, such as too much redundant data, uncertainty and complexity of the decision-making environment, difficulties of exploiting information etc. Therefore, it is a concerned topic in decision-making theoretical field about how to describe the attribute values of alternatives and reduce information loss. In qualitative environment, decision information can be usually estimated by linguistic terms (LTs) rather than exact numerical values due to universal uncertainty and the vagueness of human judgement. Zadeh [6] firstly introduced the notion of LVs). Later, Herrera and Herrera-Viedma [7, 8] proposed a linguistic assessments consensus model and further developed the steps of linguistic decision analysis. Xu [9] proposed a linguistic hybrid arithmetic average operator to solve MAGDM problems. However, these methods based on the LVs can only reflect the truth/membership degree. Then, Chen et al. [10] proposed the linguistic intuitionistic fuzzy number (LIFN) which takes the form of *γ* = (*s*_*α*_,*s*_*β*_), where *s*_*α*_ and *s*_*β*_ represent the truth/membership and falsity/non-membership degrees used by LVs based on the given LT set (LTS). It is obvious that the LIFN can describe more complex linguistic information than LVs. Based on the LIFN, some scholars [11, 12] proposed some improved aggregation operators for LIFNs, and applied them to MADM or MAGDM problems.

However, it is insufficient for LIFN to only express incomplete information, but not indeterminate and inconsistent information. In order to make up for deficiencies of LIFN, Fang and Ye [13] put forward the concept of LNN by combining LTs and simplified neutrosophic number [14–17], which consists of the truth-membership, indeterminacy-membership, and false-membership by three LVs. Compared with neutrosophic linguistic numbers (NLNs) [18–20], there is only a LV in NLNs, and the truth-membership, indeterminacy-membership, and false-membership are real values. Compared with the LIFNs, the LIFNs only reflect linguistic membership degrees and linguistic non-membership degrees in evaluation information. It cannot present the indeterminate information, which is not consistent with the ambiguity of inherent nature of human judgement. Therefore, whether NLNs or LIFNs, they can’t effectively describe attribute values of each alternative, while LNN is designed to handle the incomplete, indeterminate, and inconsistent information, and it is a generalization of LIFN and LV. Then, Fan et al. [21] proposed a LNN normalized weighted Bonferroni mean (BM) operator and a LNN normalized weighted geometric BM (LNNNWGBM) operator to handle MAGDM problems. Liang et al. [22] developed an extended TOPSIS method with LNNs to evaluate investment risks of metallic mines. Shi and Ye [23] presented a cosine similarity measure between LNNs and applied it to MAGDM problems. Ye [24] extended LNN to the linguistic neutrosophic cubic number (LNCN) and developed an MADM approach based on the LNCN weighted arithmetic averaging (LNCNWAA) operator or LNCN weighted geometric averaging (LNCNWGA) operator.

As an important tool for MADM or MAGDM, information aggregation operators have attracted wide attentions, and made a lot of achievements [25–30]. But so far, the study on the linguistic neutrosophic aggregation operators for MAGDM problems has a little progress. As we have known, there are different aggregation operators to accommodate the variety requirements. Some of them can relieve the influences of unreasonable evaluation values due to DMs’ own personal preference, such as PA operator. There are also some aggregation operators which can consider the interrelationship of the aggregated arguments, such as BM, Hamy mean (HM) operator or Maclaurin symmetric mean (MSM) operator. Qin [25] make a comparison between the HM and the MSM, the MSM is a special case of HM [31–33]. In addition, compared with BM operator, the main advantage of the HM is that it can capture the interrelationships among multi-input arguments and can provide DMs more options. However, the HM only achieved a few research results on the theory and application of inequality [34–37]. Until now, there is no research based on HM operator for aggregating incomplete, indeterminate, and inconsistent information. So it is necessary to propose some HM operators for LNNs.

From the above analysis, we can see that it’s necessary to aggregate imprecise, uncertain, and inconsistent information for the MAGDM problems with LNNs. Therefore, in order to select the best alternative(s) in practical MAGDM problems, we may need to synchronously consider the following two situations: (1) DMs usually give weight vector of attributes on the basis of their own personal preference, but they have some limited judgments for the complex decision making problems, which may have a negative effect on the final decision result. To relieve these impacts, we can utilize the entropy measure of LNS to determine the objective weights of attributes. However, the entropy measure presented by Zadeh [38] in 1965 cannot deal with LNNs. So, it is necessary to develop the entropy measure for LNNs. (2) In some practical situations, there are interrelationships among attributes and we need to capture the interrelationships among the attribute values to deal with complex decision making problems. As a result, some traditional aggregation operators, such as the BM or MSM, can be applied to reflect interactions among input arguments. However, compared with the ordinary BM, the HM can consider the interrelationship among multi-input arguments whereas the ordinary BM can only capture the interrelationship between two input arguments. On the other hand, the HM is more general than the MSM, and the MSM is a special case of HM operator. Therefore, the HM is more suitable to model interactions among input arguments than the BM and MSM.

Motivated by the above ideas, the goals of this paper are listed as follows:

1. Proposing some HM operators for LNNs, such as LNHM operator and WLNHM operator, to aggregate DMs’ incomplete, indeterminate, and inconsistent evaluation information;
2. Developing the entropy measure for LNNs;
3. Determining the object weights of the attributes to deal with incomplete weights situation under linguistic neutrosophic environment;
4. Establishing a MAGDM method based on the WLNHM operator, which provides a new method to solve multi- linguistic neutrosophic problems in actual situations;
5. Showing the advantages of the proposed approach by comparing with the existing methods [13,21,22].

The rest of this paper is organized as follows. In Sect. 2, we briefly review some basic concepts of LIFNs, LNNs and the HM operator. In Sect. 3, we propose the LNHM and WLNHM operators, we also further discuss their desirable properties and special cases. In Sect. 4, we develop an entropy measure of LNS. In Sect. 5, we propose a novel MAGDM method based on WLNNHM operator with LNNs. In Sect. 6, we compare the proposed method with those presented in [13,21,22]. In Sect. 7, we conclude the paper.

## 2. Preliminaries

### 2.1. LIFNs

**Definition 1** [10].Let *l*_*α*_,*l*_*β*_ ∈ *L* and *a* = (*l*_*α*_,*l*_*β*_), if *α* + *β* ≤ *t*, then the *a* is called a LIFN, where *l*_*α*_, *l*_*β*_ are the elements in the LTS *L* = (*l*_0_,*l*_1_,⋯,*l*_*t*_).

**Remark 1**. If *α* ∈ [0,*t*], then (*l*_*α*_,*neg*(*l*_*α*_)) is still a LIFN, where *neg*(*l*_*α*_) = *l*_*t−α*_.

**Remark 2**. We can convert the uncertain LVs [39] to the LIFNs. If $\bar{L}=\left[l_{\alpha },l_{\beta }\right]$ is an uncertain LV, where *α*,*β* ∈ [0,*t*] and *α* ≤ *β*, then we can use a LIFN (*l*_*α*_,*l*_*t−β*_) to express $\bar{L}=\left[l_{\alpha },l_{\beta }\right]$.

For convenience, we use $\Gamma_{\left[0,t\right]}^{\prime }$ to express the set of all LIFNs.

**Definition 2** [10]. Let $a=\left(l_{\alpha },l_{\beta }\right),a_{1}=\left(l_{\alpha_{1}},l_{\beta_{1}}\right),a_{2}=\left(l_{\alpha_{2}},l_{\beta_{2}}\right)\in \Gamma_{\left[0,t\right]}^{\prime }$, *λ* > 0, then the operational laws of the LIFNs are shown as follows:

$$a_{1}⊕a_{2}=\left(l_{\alpha_{1}},l_{\beta_{1}}\right)⊕\left(l_{\alpha_{2}},l_{\beta_{2}}\right)=\left(l_{\alpha_{1}+\alpha_{2}-\frac{\alpha_{1}\alpha_{2}}{t}},l_{\frac{\beta_{1}\beta_{2}}{t}}\right);$$ (1)

$$a_{1}⊗a_{2}=\left(l_{\alpha_{1}},l_{\beta_{1}}\right)⊗\left(l_{\alpha_{2}},l_{\beta_{2}}\right)=\left(l_{\frac{\alpha_{1}\alpha_{2}}{t}},l_{\beta_{1}+\beta_{2}-\frac{\beta_{1}\beta_{2}}{t}}\right);$$ (2)

$$\lambda a=\lambda \left(l_{\alpha },l_{\beta }\right)=\left(l_{t-t{\left(1-\frac{\alpha }{t}\right)}^{\lambda }},l_{t{\left(\frac{\beta }{t}\right)}^{\lambda }}\right);$$ (3)

$$a^{\lambda }={\left(l_{\alpha },l_{\beta }\right)}^{\lambda }=\left(l_{t{\left(\frac{\alpha }{t}\right)}^{\lambda }},l_{t-t{\left(1-\frac{\beta }{t}\right)}^{\lambda }}\right).$$ (4)

Obviously, the above operational results are still LIFNs.

**Definition 3** [10]. Let *a* = (*l*_*α*_,*l*_*β*_) be a LIFN based on LTS *L*. The score function and the accuracy function of the LIFN *a* are defined as follows:

S(a)=α−β; (5)

H(a)=α+β. (6)

Then, based on Definition 3, the comparison method of LIFNs is shown as follows.

**Definition 4** [10]. Let $a_{1}=\left(l_{\alpha_{1}},l_{\beta_{1}}\right),a_{2}=\left(l_{\alpha_{2}},l_{\beta_{2}}\right)\in \Gamma_{\left[0,t\right]}^{\prime }$, then

1. If *S*(*a*_1_) < *S*(*a*_2_), then *a*_1_ ≺ *a*_2_;
2. If *S*(*a*_1_) = *S*(*a*_2_),
  - and *H*(*a*_1_) < *H*(*a*_2_), then *a*_1_ ≺ *a*_2_;
  - and *H*(*a*_1_) > *H*(*a*_2_), then *a*_1_ ≻ *a*_2_.

For any two LIFNs $a_{1}=\left(l_{\alpha_{1}},l_{\beta_{1}}\right),a_{2}=\left(l_{\alpha_{2}},l_{\beta_{2}}\right)\in \Gamma_{\left[0,t\right]}^{\prime }$. If *α*_1_ ≥ *α*_2_ and *β*_1_ ≤ *β*_2_, then *α*_1_ ≥ *α*_2_.

Obviously, we have (*l*_0_,*l*_*t*_) ≤ (*l*_*α*_,*l*_*β*_) ≤ (*l*_*t*_,*l*_0_) for any $\left(l_{\alpha },l_{\beta }\right)\in \Gamma_{\left[0,t\right]}^{\prime }$.

### 2.2 LNNs

**Definition5** [13]. Let *X* be a universal set and *L* = (*l*_0_,*l*_1_,⋯,*l*_*t*_)be a LTS. A LNS *A* in *X* is characterized by a truth-membership function *α*_*A*_, a indeterminacy-membership function *β*_*A*_ and a falsity-membership function *γ*_*A*_, where *α*_*A*_,*β*_*A*_,*γ*_*A*_: *X*→[0,*t*], and ∀*x* ∈ *X*, $e=(l_{\alpha_{A}\left(x\right)},l_{\beta_{A}\left(x\right)},l_{\gamma_{A}\left(x\right)})\in A$ is called a LNN of *A*.

For convenience, we use Γ_[0,*t*]_ to express the set of all LNNs.

**Remark 3**. Let *A* be a collection of LNNs, then its complement is denoted by *A*^*C*^, which is expressed as $\alpha_{A^{C}}=\gamma_{A}$; $\beta_{A^{C}}=t-\beta_{A}$; $\gamma_{A^{C}}=\alpha_{A}$.

**Definition 6** [13]. Let $e=\left(l_{\alpha },l_{\beta },l_{\gamma }\right),e_{1}=\left(l_{\alpha_{1}},l_{\beta_{1}},l_{\gamma_{1}}\right),e_{2}=\left(l_{\alpha_{2}},l_{\beta_{2}},l_{\gamma_{2}}\right)\in \Gamma_{\left[0,t\right]}$, *λ* > 0, then the operational laws of the LNNs are shown as follows:

$$e_{1}⊕e_{2}=\left(l_{\alpha_{1}},l_{\beta_{1}},l_{\gamma_{1}}\right)⊕\left(l_{\alpha_{2}},l_{\beta_{2}},l_{\gamma_{2}}\right)=\left(l_{\alpha_{1}+\alpha_{2}-\frac{\alpha_{1}\alpha_{2}}{t}},l_{\frac{\beta_{1}\beta_{2}}{t}},l_{\frac{\gamma_{1}\gamma_{2}}{t}}\right);$$ (7)

$$e_{1}⊗e_{2}=\left(l_{\alpha_{1}},l_{\beta_{1}},l_{\gamma_{1}}\right)⊗\left(l_{\alpha_{2}},l_{\beta_{2}},l_{\gamma_{2}}\right)=\left(l_{\frac{\alpha_{1}\alpha_{2}}{t}},l_{\beta_{1}+\beta_{2}-\frac{\beta_{1}\beta_{2}}{t}},l_{\gamma_{1}+\gamma_{2}-\frac{\gamma_{1}\gamma_{2}}{t}}\right);$$ (8)

$$\lambda e=\lambda \left(l_{\alpha },l_{\beta },l_{\gamma }\right)=\left(l_{t-t{\left(1-\frac{\alpha }{t}\right)}^{\lambda }},l_{t{\left(\frac{\beta }{t}\right)}^{\lambda }},l_{t{\left(\frac{\gamma }{t}\right)}^{\lambda }}\right);$$ (9)

$$e^{\lambda }={\left(l_{\alpha },l_{\beta },l_{\gamma }\right)}^{\lambda }=\left(l_{t{\left(\frac{\alpha }{t}\right)}^{\lambda }},l_{t-t{\left(1-\frac{\beta }{t}\right)}^{\lambda }},l_{t-t{\left(1-\frac{\gamma }{t}\right)}^{\lambda }}\right).$$ (10)

It’s clearly that these operational results are still LNNs.

**Definition7** [13]. Let *e* = (*l*_*α*_,*l*_*β*_,*l*_*γ*_) be a LNN. The score function and the accuracy function of the LNN *e* are defined as follows:

$$\varphi (e)=\frac{2t+\alpha -\beta -\gamma }{3t};$$ (11)

$$\sigma (e)=\frac{\alpha -\gamma }{t}.$$ (12)

In the following, we give the comparison method of two LNNs [6].

**Definition 8** [13]. Let $e_{1}=\left(l_{\alpha_{1}},l_{\beta_{1}},l_{\gamma_{1}}\right),e_{2}=\left(l_{\alpha_{2}},l_{\beta_{2}},l_{\gamma_{2}}\right)\in \Gamma_{\left[0,t\right]}$, then

1. If *φ*(*e*_1_) < *φ*(*e*_2_), then *e*_1_ ≺ *e*_2_;
2. If *φ*(*e*_1_) = *φ*(*e*_2_),
  - and *σ*(*e*_1_) < *σ*(*e*_2_), then *e*_1_ ≺ *e*_2_;
  - and *σ*(*e*_1_) = *σ*(*e*_2_), then *e*_1_ ≈ *e*_2_.

### 2.3 Hamy mean operator

The Hamy mean (HM) [40] is proposed to capture the interrelationship among the multi-input arguments, and is defined as follows:

**Definition 9** [40] Suppose *x*_*i*_(*i* = 1,2,⋯,*n*) is a collection of nonnegative real numbers, and parameter *k* = 1,2,⋯,*n*. The HM is defined as

$$HM^{\left(k\right)}\left(x_{1},x_{2},\cdots ,x_{n}\right)=\frac{\sum_{1\leq i_{1}<\cdots <i_{k}\leq n}{\left(\prod_{j=1}^{k}x_{i_{j}}\right)}^{1/k}}{\left(\begin{array}{c} n\\ k \end{array}\right)}$$ (13)

where (*i*_1_,*i*_2_,⋯,*i*_*k*_)traverses all the *k-tuple* combination of (1,2,⋯,*n*) and $\left(\begin{array}{c} n\\ k \end{array}\right)$ is the binomial coefficient, and $\left(\begin{array}{c} n\\ k \end{array}\right)=\frac{n!}{k!\left(n-k\right)!}$.

Obviously, the HM has the following properties:

1. *HM*^(*k*)^ (0,0,⋯,0) = 0, *HM*^(*k*)^ (*x*,*x*,⋯,*x*) = *x*;
2. *HM*^(*k*)^ (*x*_1_,*x*_2_,⋯,*x*_*n*_) ≤ *HM*^(*k*)^ (*y*_1_,*y*_2_,⋯,*y*_*n*_), if *x*_*i*_ ≤ *y*_*i*_ for all *i*;
3. min{*x*_*i*_} ≤ *HM*^(*k*)^ (*x*_1_,*x*_2_,⋯,*x*_*n*_) ≤ max{*x*_*i*_}

## 3. Linguistic neutrosophic HM aggregation operators

In this section, based on the operational laws of LNNs, we shall explore the HM operator to deal with LNNs and develop LNHM operator and WLNHM operator, and then we also discuss some properties and some special cases of these new operators.

### 3.1 LNHM operator

**Definition 10.** Let *e*_*i*_ (*i* = 1,2,⋯,*n*) be a collection of LNNs, the LNHM operator is defined as follows:

$$LNHM^{\left(k\right)}(e_{1},e_{2},\ldots .,e_{n})=\frac{\sum_{1\leq i_{1}<\cdots <i_{k}\leq n}{\left(\prod_{j=1}^{k}e_{i_{j}}\right)}^{\frac{1}{k}}}{\left(\begin{array}{c} n\\ k \end{array}\right)}$$ (14)

where (*i*_1_,*i*_2_,⋯,*i*_*k*_) traverses all the *k-tuple* combination of (1,2,⋯,*n*) and $\left(\begin{array}{c} n\\ k \end{array}\right)$ is the binomial coefficient, and $\left(\begin{array}{c} n\\ k \end{array}\right)=\frac{n!}{k!\left(n-k\right)!}$.

**Theorem 1.** Let $e_{i}=\left(l_{\alpha_{i}},l_{\beta_{i}},l_{\gamma_{i}}\right)$ (*i* = 1,2,⋯,*n*) be a collection of LNNs, then the aggregated value from definition 10 is still a LNN, and

$$LNHM^{\left(k\right)}(e_{1},e_{2},\ldots .,e_{n})=\left(l_{t-t{\left(\prod_{1\leq i_{1}<\cdots <i_{k}\leq n}\left(1-{\left(\frac{\prod_{j=1}^{k}\alpha_{i_{j}}}{t^{k}}\right)}^{\frac{1}{k}}\right)\right)}^{\frac{1}{\left(\begin{array}{c} n\\ k \end{array}\right)}}},l_{t{\left(\prod_{1\leq i_{1}<\cdots <i_{k}\leq n}\left(1-{\left(\prod_{j=1}^{k}\left(1-\frac{\beta_{i_{j}}}{t}\right)\right)}^{\frac{1}{k}}\right)\right)}^{{}^{\frac{1}{\left(\begin{array}{c} n\\ k \end{array}\right)}}}},l_{t{\left(\prod_{1\leq i_{1}<\cdots <i_{k}\leq n}\left(1-{\left(\prod_{j=1}^{k}\left(1-\frac{\gamma_{i_{j}}}{t}\right)\right)}^{\frac{1}{k}}\right)\right)}^{{}^{\frac{1}{\left(\begin{array}{c} n\\ k \end{array}\right)}}}}\right)$$ (15)

Proof.

According to Eqs (7)–(10), we have

$$\prod_{j=1}^{k}e_{i_{j}}=\left(l_{\frac{\prod_{j=1}^{k}\alpha_{i_{j}}}{t^{k-1}}},l_{t-t\prod_{j=1}^{k}\left(1-\frac{\beta_{i_{j}}}{t}\right)},l_{t-t\prod_{j=1}^{k}\left(1-\frac{\gamma_{i_{j}}}{t}\right)}\right)$$

and

$$\begin{array}{l} {\left(\prod_{j=1}^{k}e_{i_{j}}\right)}^{\frac{1}{k}}=\left(l_{t{\left(\frac{\prod_{j=1}^{k}\alpha_{i_{j}}}{t^{k}}\right)}^{\frac{1}{k}}},l_{t-t{\left(\prod_{j=1}^{k}\left(1-\frac{\beta_{i_{j}}}{t}\right)\right)}^{\frac{1}{k}}},l_{t-t{\left(\prod_{j=1}^{k}\left(1-\frac{\gamma_{i_{j}}}{t}\right)\right)}^{\frac{1}{k}}}\right)\\ \sum_{1\leq i_{1}<\cdots <i_{k}\leq n}{\left(\prod_{j=1}^{k}e_{i_{j}}\right)}^{\frac{1}{k}}=\left(l_{t-t\prod_{1\leq i_{1}<\cdots <i_{k}\leq n}\left(1-{\left(\frac{\prod_{j=1}^{k}\alpha_{i_{j}}}{t^{k}}\right)}^{\frac{1}{k}}\right)},l_{t\prod_{1\leq i_{1}<\cdots <i_{k}\leq n}\left(1-{\left(\prod_{j=1}^{k}\left(1-\frac{\beta_{i_{j}}}{t}\right)\right)}^{\frac{1}{k}}\right)},l_{t\prod_{1\leq i_{1}<\cdots <i_{k}\leq n}\left(1-{\left(\prod_{j=1}^{k}\left(1-\frac{\gamma_{i_{j}}}{t}\right)\right)}^{\frac{1}{k}}\right)}\right) \end{array}$$

Then we obtain

$$\frac{1}{\left(\begin{array}{c} n\\ k \end{array}\right)}\sum_{1\leq i_{1}<\cdots <i_{k}\leq n}{\left(\prod_{j=1}^{k}e_{i_{j}}\right)}^{\frac{1}{k}}=\left(l_{t-t{\left(\prod_{1\leq i_{1}<\cdots <i_{k}\leq n}\left(1-{\left(\frac{\prod_{j=1}^{k}\alpha_{i_{j}}}{t^{k}}\right)}^{\frac{1}{k}}\right)\right)}^{\frac{1}{\left(\begin{array}{c} n\\ k \end{array}\right)}}},l_{t{\left(\prod_{1\leq i_{1}<\cdots <i_{k}\leq n}\left(1-{\left(\prod_{j=1}^{k}\left(1-\frac{\beta_{i_{j}}}{t}\right)\right)}^{\frac{1}{k}}\right)\right)}^{{}^{\frac{1}{\left(\begin{array}{c} n\\ k \end{array}\right)}}}},l_{t{\left(\prod_{1\leq i_{1}<\cdots <i_{k}\leq n}\left(1-{\left(\prod_{j=1}^{k}\left(1-\frac{\gamma_{i_{j}}}{t}\right)\right)}^{\frac{1}{k}}\right)\right)}^{{}^{\frac{1}{\left(\begin{array}{c} n\\ k \end{array}\right)}}}}\right)$$

Therefore,

$$LNHM^{\left(k\right)}(e_{1},e_{2},\ldots .,e_{n})=\left(l_{t-t{\left(\prod_{1\leq i_{1}<\cdots <i_{k}\leq n}\left(1-{\left(\frac{\prod_{j=1}^{k}\alpha_{i_{j}}}{t^{k}}\right)}^{\frac{1}{k}}\right)\right)}^{\frac{1}{\left(\begin{array}{c} n\\ k \end{array}\right)}}},l_{t{\left(\prod_{1\leq i_{1}<\cdots <i_{k}\leq n}\left(1-{\left(\prod_{j=1}^{k}\left(1-\frac{\beta_{i_{j}}}{t}\right)\right)}^{\frac{1}{k}}\right)\right)}^{{}^{\frac{1}{\left(\begin{array}{c} n\\ k \end{array}\right)}}}},l_{t{\left(\prod_{1\leq i_{1}<\cdots <i_{k}\leq n}\left(1-{\left(\prod_{j=1}^{k}\left(1-\frac{\gamma_{i_{j}}}{t}\right)\right)}^{\frac{1}{k}}\right)\right)}^{{}^{\frac{1}{\left(\begin{array}{c} n\\ k \end{array}\right)}}}}\right).$$

In addition, since $\begin{array}{l} 0\leq t-t{\left(\prod_{1\leq i_{1}<\cdots <i_{k}\leq n}\left(1-{\left(\frac{\prod_{j=1}^{k}\alpha_{i_{j}}}{t^{k}}\right)}^{\frac{1}{k}}\right)\right)}^{\frac{1}{\left(\begin{array}{c} n\\ k \end{array}\right)}}\leq t \end{array}$,

$$\begin{array}{l} 0\leq t{\left(\prod_{1\leq i_{1}<\cdots <i_{k}\leq n}\left(1-{\left(\prod_{j=1}^{k}\left(1-\frac{\beta_{i_{j}}}{t}\right)\right)}^{\frac{1}{k}}\right)\right)}^{{}^{\frac{1}{\left(\begin{array}{c} n\\ k \end{array}\right)}}}\leq t,\;0\leq t{\left(\prod_{1\leq i_{1}<\cdots <i_{k}\leq n}\left(1-{\left(\prod_{j=1}^{k}\left(1-\frac{\gamma_{i_{j}}}{t}\right)\right)}^{\frac{1}{k}}\right)\right)}^{{}^{\frac{1}{\left(\begin{array}{c} n\\ k \end{array}\right)}}}\leq t, \end{array}$$

So, $\left(l_{t-t{\left(\prod_{1\leq i_{1}<\cdots <i_{k}\leq n}\left(1-{\left(\frac{\prod_{j=1}^{k}\alpha_{i_{j}}}{t^{k}}\right)}^{\frac{1}{k}}\right)\right)}^{\frac{1}{\left(\begin{array}{c} n\\ k \end{array}\right)}}},l_{t{\left(\prod_{1\leq i_{1}<\cdots <i_{k}\leq n}\left(1-{\left(\prod_{j=1}^{k}\left(1-\frac{\beta_{i_{j}}}{t}\right)\right)}^{\frac{1}{k}}\right)\right)}^{{}^{\frac{1}{\left(\begin{array}{c} n\\ k \end{array}\right)}}}},l_{t{\left(\prod_{1\leq i_{1}<\cdots <i_{k}\leq n}\left(1-{\left(\prod_{j=1}^{k}\left(1-\frac{\gamma_{i_{j}}}{t}\right)\right)}^{\frac{1}{k}}\right)\right)}^{{}^{\frac{1}{\left(\begin{array}{c} n\\ k \end{array}\right)}}}}\right)$ is also a LNN, which Theorem1 is proved.

**Example 3.1** Let *L* = {*l*_0_ = extremely low, *l*_1_ = very low, *l*_2_ = low, *l*_3_ = fair, *l*_4_ = high, *l*_5_ = very high, *l*_6_ = extremely high} and *e*_1_ = (*l*_3_,*l*_2_,*l*_1_), *e*_2_ = (*l*_6_,*l*_4_,*l*_2_), *e*_3_ = (*l*_5_,*l*_1_,*l*_3_), *e*_4_ = (*l*_5_,*l*_4_,*l*_3_), be four LNNs based on *L*.Then, we can use the proposed LNHM operator to aggregate these four LNNs (suppose *k* = 2), and generate a comprehensive value *LNHM*^(*k*)^(*e*_1_,*e*_2_,*e*_3_,*e*_4_) = (*l*_*α*_,*l*_*β*_,*l*_*γ*_), described as follows.

- $\frac{1}{\left(\begin{array}{c} n\\ k \end{array}\right)}=\frac{k!\left(n-k\right)!}{n!}=\frac{2!\left(4-2\right)!}{4!}=\frac{1}{6};$
- $t-t{\left(\prod_{1\leq i_{1}<\cdots <i_{k}\leq n}\left(1-{\left(\frac{\prod_{j=1}^{k}\alpha_{i_{j}}}{t^{k}}\right)}^{\frac{1}{k}}\right)\right)}^{\frac{1}{\left(\begin{array}{c} n\\ k \end{array}\right)}}=6-6\times {\left(\left(1-{\left(\frac{3\times 6}{6^{2}}\right)}^{\frac{1}{2}}\right)\times \left(1-{\left(\frac{3\times 5}{6^{2}}\right)}^{\frac{1}{2}}\right)\times \left(1-{\left(\frac{3\times 5}{6^{2}}\right)}^{\frac{1}{2}}\right)\times \left(1-{\left(\frac{6\times 5}{6^{2}}\right)}^{\frac{1}{2}}\right)\times \left(1-{\left(\frac{6\times 5}{6^{2}}\right)}^{\frac{1}{2}}\right)\times \left(1-{\left(\frac{5\times 5}{6^{2}}\right)}^{\frac{1}{2}}\right)\right)}^{\frac{1}{6}}=4.8619;$
- $t{\left(\prod_{1\leq i_{1}<\cdots <i_{k}\leq n}\left(1-{\left(\prod_{j=1}^{k}\left(1-\frac{\beta_{i_{j}}}{t}\right)\right)}^{\frac{1}{k}}\right)\right)}^{\frac{1}{\left(\begin{array}{c} n\\ k \end{array}\right)}}=6\times {\left(\left(1-{\left(\left(1-\frac{2}{6}\right)\times \left(1-\frac{4}{6}\right)\right)}^{\frac{1}{2}}\right)\times \left(1-{\left(\left(1-\frac{2}{6}\right)\times \left(1-\frac{1}{6}\right)\right)}^{\frac{1}{2}}\right)\times \left(1-{\left(\left(1-\frac{2}{6}\right)\times \left(1-\frac{4}{6}\right)\right)}^{\frac{1}{2}}\right)\times \left(1-{\left(\left(1-\frac{4}{6}\right)\times \left(1-\frac{1}{6}\right)\right)}^{\frac{1}{2}}\right)\times \left(1-{\left(\left(1-\frac{4}{6}\right)\times \left(1-\frac{4}{6}\right)\right)}^{\frac{1}{2}}\right)\times \left(1-{\left(\left(1-\frac{1}{6}\right)\times \left(1-\frac{4}{6}\right)\right)}^{\frac{1}{2}}\right)\right)}^{\frac{1}{6}}=2.8126;$
- $t{\left(\prod_{1\leq i_{1}<\cdots <i_{k}\leq n}\left(1-{\left(\prod_{j=1}^{k}\left(1-\frac{\gamma_{i_{j}}}{t}\right)\right)}^{\frac{1}{k}}\right)\right)}^{\frac{1}{\left(\begin{array}{c} n\\ k \end{array}\right)}}=6\times {\left(\left(1-{\left(\left(1-\frac{1}{6}\right)\times \left(1-\frac{2}{6}\right)\right)}^{\frac{1}{2}}\right)\times \left(1-{\left(\left(1-\frac{1}{6}\right)\times \left(1-\frac{3}{6}\right)\right)}^{\frac{1}{2}}\right)\times \left(1-{\left(\left(1-\frac{1}{6}\right)\times \left(1-\frac{3}{6}\right)\right)}^{\frac{1}{2}}\right)\times \left(1-{\left(\left(1-\frac{2}{6}\right)\times \left(1-\frac{3}{6}\right)\right)}^{\frac{1}{2}}\right)\times \left(1-{\left(\left(1-\frac{2}{6}\right)\times \left(1-\frac{3}{6}\right)\right)}^{\frac{1}{2}}\right)\times \left(1-{\left(\left(1-\frac{3}{6}\right)\times \left(1-\frac{3}{6}\right)\right)}^{\frac{1}{2}}\right)\right)}^{\frac{1}{6}}=2.2603$

Therefore, we can obtain *LNHM*^(2)^(*e*_1_,*e*_2_,*e*_3_,*e*_4_) = (*l*_*α*_,*l*_*β*_,*l*_*γ*_) = (*l*_4.8619_,*l*_2.8126_,*l*_2.2603_).

In what follows, we shall investigate some desirable properties of LNNs.

**Property 1** (Idempotency). If *e*_*i*_ = *e* = (*l*_*α*_,*l*_*β*_,*l*_*γ*_) for all (*i* = 1,2,⋯,*n*), then

$$LNHM^{\left(k\right)}(e,e,\ldots .,e)=\left(l_{\alpha },l_{\beta },l_{\gamma }\right)$$ (16)

Proof.

Since *e* = (*l*_*α*_,*l*_*β*_,*l*_*γ*_), based on Theorem 1, we have

$$\begin{array}{l} LNHM^{\left(k\right)}(e,e,\ldots .,e)=\left(l_{t-t{\left(\prod_{1\leq i_{1}<\cdots <i_{k}\leq n}\left(1-{\left(\frac{\alpha^{k}}{t^{k}}\right)}^{\frac{1}{k}}\right)\right)}^{\frac{1}{\left(\begin{array}{c} n\\ k \end{array}\right)}}},l_{t{\left(\prod_{1\leq i_{1}<\cdots <i_{k}\leq n}\left(1-{\left({\left(1-\frac{\beta }{t}\right)}^{k}\right)}^{\frac{1}{k}}\right)\right)}^{\frac{1}{\left(\begin{array}{c} n\\ k \end{array}\right)}}},l_{t{\left(\prod_{1\leq i_{1}<\cdots <i_{k}\leq n}\left(1-{\left({\left(1-\frac{\gamma }{t}\right)}^{k}\right)}^{\frac{1}{k}}\right)\right)}^{\frac{1}{\left(\begin{array}{c} n\\ k \end{array}\right)}}}\right)\\ =\left(l_{t-t{\left({\left(1-\frac{\alpha }{t}\right)}^{\left(\begin{array}{c} n\\ k \end{array}\right)}\right)}^{\frac{1}{\left(\begin{array}{c} n\\ k \end{array}\right)}}},l_{t{\left({\left(1-\left(1-\frac{\beta }{t}\right)\right)}^{\left(\begin{array}{c} n\\ k \end{array}\right)}\right)}^{\frac{1}{\left(\begin{array}{c} n\\ k \end{array}\right)}}},l_{t{\left({\left(1-\left(1-\frac{\gamma }{t}\right)\right)}^{\left(\begin{array}{c} n\\ k \end{array}\right)}\right)}^{\frac{1}{\left(\begin{array}{c} n\\ k \end{array}\right)}}}\right)=\left(l_{t-t\left(1-\frac{\alpha }{t}\right)},l_{t\left(1-\left(1-\frac{\beta }{t}\right)\right)},l_{t\left(1-\left(1-\frac{\gamma }{t}\right)\right)}\right)\\ =\left(l_{\alpha },l_{\beta },l_{\gamma }\right)=e. \end{array}$$

**Property 2** (Commutativity). Let *e*_*i*_ (*i* = 1,2,⋯,*n*) be a collection of LNNs, and $\left(e_{1}^{\prime },e_{2}^{\prime },\ldots .,e_{n}^{\prime }\right)$ be any permutation of (*e*_1_,*e*_2_,….,*e*_*n*_), then

$$LNHM^{\left(k\right)}(e_{1}^{\prime },e_{2}^{\prime },\ldots .,e_{n}^{\prime })=LNHM^{\left(k\right)}(e_{1},e_{2},\ldots .,e_{n})$$ (17)

Proof.

Based on Definition 10, the conclusion is obvious.

$$LNHM^{\left(k\right)}(e_{1}^{\prime },e_{2}^{\prime },\ldots .,e_{n}^{\prime })=\frac{\sum_{1\leq i_{1}<\cdots <i_{k}\leq n}{\left(\prod_{j=1}^{k}e_{i_{j}}^{\prime }\right)}^{\frac{1}{k}}}{\left(\begin{array}{c} n\\ k \end{array}\right)}=\frac{\sum_{1\leq i_{1}<\cdots <i_{k}\leq n}{\left(\prod_{j=1}^{k}e_{i_{j}}\right)}^{\frac{1}{k}}}{\left(\begin{array}{c} n\\ k \end{array}\right)}=LNHM^{\left(k\right)}(e_{1},e_{2},\ldots .,e_{n}).$$

**Property 3** (Monotonicity). Let $e_{i}=\left(l_{\alpha_{i}},l_{\beta_{i}},l_{\gamma_{i}}\right)$, $f_{i}=\left(l_{p_{i}},l_{q_{i}},l_{r_{i}}\right)$ (*i* = 1,2,…,*n*) be two collections of LNNs, if *α*_*i*_ ≤ *p*_*i*_, *β*_*i*_ ≥ *q*_*i*_, *γ*_*i*_ ≥ *r*_*i*_ for all *i*, then

$$LNHM^{\left(k\right)}(e_{1},e_{2},\ldots ,e_{n})\leq LNHM^{\left(k\right)}(f_{1},f_{2},\ldots ,f_{n})$$ (18)

Proof.

Since 0 ≤ *α*_*i*_ ≤ *p*_*i*_, *β*_*i*_ ≥ *q*_*i*_ ≥ 0, *γ*_*i*_ ≥ *r*_*i*_ ≥ 0, *t* ≥ 0, and according to Theorem 1, we can obtain

$$t-t{\left(\prod_{1\leq i_{1}<\cdots <i_{k}\leq n}\left(1-{\left(\frac{\prod_{j=1}^{k}\alpha_{i_{j}}}{t^{k}}\right)}^{\frac{1}{k}}\right)\right)}^{\frac{1}{\left(\begin{array}{c} n\\ k \end{array}\right)}}\leq t-t{\left(\prod_{1\leq i_{1}<\cdots <i_{k}\leq n}\left(1-{\left(\frac{\prod_{j=1}^{k}p_{i_{j}}}{t^{k}}\right)}^{\frac{1}{k}}\right)\right)}^{\frac{1}{\left(\begin{array}{c} n\\ k \end{array}\right)}},$$

$$-t{\left(\prod_{1\leq i_{1}<\cdots <i_{k}\leq n}\left(1-{\left(\prod_{j=1}^{k}\left(1-\frac{\beta_{i_{j}}}{t}\right)\right)}^{\frac{1}{k}}\right)\right)}^{{}^{\frac{1}{\left(\begin{array}{c} n\\ k \end{array}\right)}}}\leq -t{\left(\prod_{1\leq i_{1}<\cdots <i_{k}\leq n}\left(1-{\left(\prod_{j=1}^{k}\left(1-\frac{q_{i_{j}}}{t}\right)\right)}^{\frac{1}{k}}\right)\right)}^{{}^{\frac{1}{\left(\begin{array}{c} n\\ k \end{array}\right)}}},$$

$$-t{\left(\prod_{1\leq i_{1}<\cdots <i_{k}\leq n}\left(1-{\left(\prod_{j=1}^{k}\left(1-\frac{\gamma_{i_{j}}}{t}\right)\right)}^{\frac{1}{k}}\right)\right)}^{{}^{\frac{1}{\left(\begin{array}{c} n\\ k \end{array}\right)}}}\leq -t{\left(\prod_{1\leq i_{1}<\cdots <i_{k}\leq n}\left(1-{\left(\prod_{j=1}^{k}\left(1-\frac{r_{i_{j}}}{t}\right)\right)}^{\frac{1}{k}}\right)\right)}^{{}^{\frac{1}{\left(\begin{array}{c} n\\ k \end{array}\right)}}}.$$

Let *e* = *LNHM*^(*k*)^(*e*_1_,*e*_2_,…,*e*_*n*_), *f* = *LNHM*^(*k*)^(*f*_1_,*f*_2_,…,*f*_*n*_) and *φ*(*e*),*φ*(*f*) be the score functions of *e* and *f*. According to the score value in Eq (11) and the above inequality, we can imply *φ*(*e*) ≤ *φ*(*f*). Then we discuss the following cases:

1. if *φ*(*e*) ≤ *φ*(*f*), we can get *LNHM*^(*k*)^(*e*_1_,*e*_2_,…,*e*_*n*_) ≤ *LNHM*^(*k*)^(*f*_1_,*f*_2_,…,*f*_*n*_);
2. if *φ*(*e*) = *φ*(*f*), then

$$\begin{array}{l} \frac{2t+t-t{\left(\prod_{1\leq i_{1}<\cdots <i_{k}\leq n}\left(1-{\left(\frac{\prod_{j=1}^{k}\alpha_{i_{j}}}{t^{k}}\right)}^{\frac{1}{k}}\right)\right)}^{\frac{1}{\left(\begin{array}{c} n\\ k \end{array}\right)}}-t{\left(\prod_{1\leq i_{1}<\cdots <i_{k}\leq n}\left(1-{\left(\prod_{j=1}^{k}\left(1-\frac{\beta_{i_{j}}}{t}\right)\right)}^{\frac{1}{k}}\right)\right)}^{{}^{\frac{1}{\left(\begin{array}{c} n\\ k \end{array}\right)}}}-t{\left(\prod_{1\leq i_{1}<\cdots <i_{k}\leq n}\left(1-{\left(\prod_{j=1}^{k}\left(1-\frac{\gamma_{i_{j}}}{t}\right)\right)}^{\frac{1}{k}}\right)\right)}^{{}^{\frac{1}{\left(\begin{array}{c} n\\ k \end{array}\right)}}}}{3t}=\\ \frac{2t+t-t{\left(\prod_{1\leq i_{1}<\cdots <i_{k}\leq n}\left(1-{\left(\frac{\prod_{j=1}^{k}p_{i_{j}}}{t^{k}}\right)}^{\frac{1}{k}}\right)\right)}^{\frac{1}{\left(\begin{array}{c} n\\ k \end{array}\right)}}-t{\left(\prod_{1\leq i_{1}<\cdots <i_{k}\leq n}\left(1-{\left(\prod_{j=1}^{k}\left(1-\frac{q_{i_{j}}}{t}\right)\right)}^{\frac{1}{k}}\right)\right)}^{{}^{\frac{1}{\left(\begin{array}{c} n\\ k \end{array}\right)}}}-t{\left(\prod_{1\leq i_{1}<\cdots <i_{k}\leq n}\left(1-{\left(\prod_{j=1}^{k}\left(1-\frac{r_{i_{j}}}{t}\right)\right)}^{\frac{1}{k}}\right)\right)}^{{}^{\frac{1}{\left(\begin{array}{c} n\\ k \end{array}\right)}}}}{3t} \end{array}$$

Since 0 ≤ *α*_*i*_ ≤ *p*_*i*_, *β*_*i*_ ≥ *q*_*i*_ ≥ 0, *γ*_*i*_ ≥ *r*_*i*_ ≥, we can deduce that

$$t-t{\left(\prod_{1\leq i_{1}<\cdots <i_{k}\leq n}\left(1-{\left(\frac{\prod_{j=1}^{k}\alpha_{i_{j}}}{t^{k}}\right)}^{\frac{1}{k}}\right)\right)}^{\frac{1}{\left(\begin{array}{c} n\\ k \end{array}\right)}}=t-t{\left(\prod_{1\leq i_{1}<\cdots <i_{k}\leq n}\left(1-{\left(\frac{\prod_{j=1}^{k}p_{i_{j}}}{t^{k}}\right)}^{\frac{1}{k}}\right)\right)}^{\frac{1}{\left(\begin{array}{c} n\\ k \end{array}\right)}},$$

$$-t{\left(\prod_{1\leq i_{1}<\cdots <i_{k}\leq n}\left(1-{\left(\prod_{j=1}^{k}\left(1-\frac{\gamma_{i_{j}}}{t}\right)\right)}^{\frac{1}{k}}\right)\right)}^{{}^{\frac{1}{\left(\begin{array}{c} n\\ k \end{array}\right)}}}=-t{\left(\prod_{1\leq i_{1}<\cdots <i_{k}\leq n}\left(1-{\left(\prod_{j=1}^{k}\left(1-\frac{r_{i_{j}}}{t}\right)\right)}^{\frac{1}{k}}\right)\right)}^{{}^{\frac{1}{\left(\begin{array}{c} n\\ k \end{array}\right)}}},$$

and based on the accuracy value in Eq (12), there is *σ*(*e*) = *σ*(*f*). So, finally, we have *LNHM*^(*k*)^(*e*_1_,*e*_2_,…,*e*_*n*_) ≤ *LNHM*^(*k*)^(*f*_1_,*f*_2_,…,*f*_*n*_)

**Property 4 (**Boundedness). Let $e_{i}=\left(l_{\alpha_{i}},l_{\beta_{i}},l_{\gamma_{i}}\right)$ (*i* = 1,2,⋯,*n*) be a collection of LNNs, and $e^{+}=\max(e_{1},e_{2},\ldots .,e_{n})=\left(l_{\max(\alpha_{i})},l_{\min(\beta_{i})},l_{\min(\gamma_{i})}\right)$, $e^{-}=\min(e_{1},e_{2},\ldots .,e_{n})=\left(l_{\min(\alpha_{i})},l_{\max(\beta_{i})},l_{\max(\gamma_{i})}\right)$, then

$$e^{-}\leq LNHM^{\left(k\right)}(e_{1},e_{2},\ldots .,e_{n})\leq e^{+}$$ (19)

Proof.

Based on Properties 1 and 3, we have

$$LNHM^{\left(k\right)}(e_{1},e_{2},\ldots .,e_{n})\geq LNHM^{\left(k\right)}\left(e_{1}{}^{-},e_{2}{}^{-},\ldots ,e_{n}{}^{-}\right)=e^{-},$$

$$LNHM^{\left(k\right)}(e_{1},e_{2},\ldots .,e_{n})\leq LNHM^{\left(k\right)}\left(e_{1}^{+},e_{2}^{+},\ldots ,e_{n}^{+}\right)=e^{+}.$$

Thus the proof is completed.

Further, we will discuss some specials of the LNHM operator with respect to the parameter *k*.

1. When *k* = 1, the LNHM operator in (14) will reduce to the LNA (Linguistic Neutrosophic Averaging) operator
  $$\begin{array}{l} LNHM^{\left(1\right)}(e_{1},e_{2},\ldots .,e_{n})=\frac{\sum_{1\leq i_{1}\leq n}{\left(\prod_{j=1}^{1}e_{i_{j}}\right)}^{\frac{1}{1}}}{\left(\begin{array}{c} n\\ 1 \end{array}\right)}\\ =\left(l_{t-t{\left(\prod_{1\leq i_{1}\leq n}\left(1-{\left(\prod_{j=1}^{1}\alpha_{i_{j}}\right)}^{\frac{1}{1}}\right)\right)}^{\frac{1}{\left(\begin{array}{c} n\\ 1 \end{array}\right)}}},l_{t{\left(\prod_{1\leq i_{1}\leq n}\left(1-{\left(\prod_{j=1}^{1}\left(1-\frac{\beta_{i_{j}}}{t}\right)\right)}^{\frac{1}{1}}\right)\right)}^{\frac{1}{\left(\begin{array}{c} n\\ 1 \end{array}\right)}}},l_{t{\left(\prod_{1\leq i_{1}\leq n}\left(1-{\left(\prod_{j=1}^{1}\left(1-\frac{\gamma_{i_{j}}}{t}\right)\right)}^{\frac{1}{1}}\right)\right)}^{\frac{1}{\left(\begin{array}{c} n\\ 1 \end{array}\right)}}}\right)\\ =\left(l_{t-t{\left(\prod_{i=1}^{n}\left(1-\alpha_{i}\right)\right)}^{\frac{1}{n}}},l_{t{\left(\prod_{j=1}^{n}\frac{\beta_{i}}{t}\right)}^{\frac{1}{n}}},l_{t{\left(\prod_{j=1}^{n}\frac{\gamma_{i}}{t}\right)}^{\frac{1}{n}}}\right)\left(leti_{1}=i\right)=\frac{1}{n}\sum_{i=1}^{n}e_{i}=LNA\left(e_{1},e_{2},\ldots ,e_{n}\right) \end{array}$$ (20)
2. When *k* = *n*, the LNHM operator in (14) will reduce to the LNG (Linguistic Neutrosophic Geometric) operator
  $$\begin{array}{l} LNHM^{\left(n\right)}(e_{1},e_{2},\ldots .,e_{n})=\frac{\sum_{1\leq i_{1}<\cdots i_{k}\leq n}{\left(\prod_{j=1}^{n}e_{i_{j}}\right)}^{\frac{1}{n}}}{\left(\begin{array}{c} n\\ n \end{array}\right)}\\ =\left(l_{t-t{\left(\prod_{1\leq i_{1}<\cdots i_{k}\leq n}\left(1-{\left(\frac{\prod_{j=1}^{n}\alpha_{i_{j}}}{t^{n}}\right)}^{\frac{1}{n}}\right)\right)}^{\frac{1}{\left(\begin{array}{c} n\\ n \end{array}\right)}}},l_{t{\left(\prod_{1\leq i_{1}<\cdots i_{k}\leq n}\left(1-{\left(\prod_{j=1}^{n}\left(1-\frac{\beta_{i_{j}}}{t}\right)\right)}^{\frac{1}{n}}\right)\right)}^{\frac{1}{\left(\begin{array}{c} n\\ n \end{array}\right)}}},l_{t{\left(\prod_{1\leq i_{1}<\cdots i_{k}\leq n}\left(1-{\left(\prod_{j=1}^{n}\left(1-\frac{\gamma_{i_{j}}}{t}\right)\right)}^{\frac{1}{n}}\right)\right)}^{\frac{1}{\left(\begin{array}{c} n\\ n \end{array}\right)}}}\right)\\ =\left(l_{t-t\left(1-{\left(\frac{\prod_{j=1}^{n}\alpha_{i_{j}}}{t^{n}}\right)}^{\frac{1}{n}}\right)},l_{t\left(1-{\left(\prod_{j=1}^{n}\left(1-\frac{\beta_{i_{j}}}{t}\right)\right)}^{\frac{1}{n}}\right)},l_{t\left(1-{\left(\prod_{j=1}^{n}\left(1-\frac{\gamma_{i_{j}}}{t}\right)\right)}^{\frac{1}{n}}\right)}\right)\\ =\left(l_{t{\left(\frac{\prod_{i=1}^{n}\alpha_{i}}{t^{n}}\right)}^{\frac{1}{n}}},l_{t-t{\left(\prod_{i=1}^{n}\left(1-\frac{\beta_{i}}{t}\right)\right)}^{\frac{1}{n}}},l_{t-t{\left(\prod_{j=1}^{n}\left(1-\frac{\gamma_{i_{j}}}{t}\right)\right)}^{\frac{1}{n}}}\right)\left(let\;i_{j}=i\right)=\prod_{i=1}^{n}e_{i}^{\frac{1}{n}}=LNG\left(e_{1},e_{2},\ldots ,e_{n}\right). \end{array}$$ (21)

### 3.2 WLNHM operator

**Definition 11.** Let *e*_*i*_ (*i* = 1,2,⋯,*n*) be a collection of LNs, *ω* = (*ω*_1_,*ω*_2_,….,*ω*_*n*_)^*T*^ be the weight vector of ${\tilde{l}}_{i}$, with *ω*_*i*_ ∈ [0,1] and $\sum_{i=1}^{n}\omega_{i}=1$, then we can define the WLNHM operator as follows.

$$WLNHM^{\left(k\right)}(e_{1},e_{2},\ldots .,e_{n})=\frac{\sum_{1\leq i_{1}<\cdots <i_{k}\leq n}{\left(\prod_{j=1}^{k}w_{i_{j}}e_{i_{j}}\right)}^{\frac{1}{k}}}{\left(\begin{array}{c} n\\ k \end{array}\right)}$$ (22)

where (*i*_1_,*i*_2_,….,*i*_*k*_) traverses all the *k*–*tuple* combination of (1,2,….,*n*), and $\left(\begin{array}{c} n\\ k \end{array}\right)$ is the binomial coefficient, and $\left(\begin{array}{c} n\\ k \end{array}\right)=\frac{n!}{k!\left(n-k\right)!}$.

Based on the operational rules of LNNs presented in Eqs (7)–(10), from Eq (22), we can derive the following theorem.

**Theorem 3.** Let $e_{i}=\left(l_{\alpha_{i}},l_{\beta_{i}},l_{\gamma_{i}}\right)$ (*i* = 1,2,⋯,*n*) be a collection of LNNs, *ω* = (*ω*_1_,*ω*_2_,….,*ω*_*n*_)^*T*^ be the weight vector of *e*_*i*_ with *ω*_*i*_ ∈ [0,1], *i* = 1,2,….,*n* and $\sum_{i=1}^{n}\omega_{i}=1$. Then the aggregated value obtained from the WLNHM operator in (22) is also a LNN, and

$$\begin{array}{l} WLNHM^{\left(k\right)}(e_{1},e_{2},\ldots .,e_{n})=\\ \left(l_{t-t{\left(\prod_{1\leq i_{1}<\cdots <i_{k}\leq n}\left(1-{\left(\prod_{j=1}^{k}\left(1-{\left(1-\frac{\alpha_{i_{j}}}{t}\right)}^{w_{i_{j}}}\right)\right)}^{\frac{1}{k}}\right)\right)}^{\frac{1}{\left(\begin{array}{c} n\\ k \end{array}\right)}}},l_{t{\left(\prod_{1\leq i_{1}<\cdots <i_{k}\leq n}\left(1-{\left(\prod_{j=1}^{k}\left(1-{\left(\frac{\beta_{i_{j}}}{t}\right)}^{w_{i_{j}}}\right)\right)}^{\frac{1}{k}}\right)\right)}^{\frac{1}{\left(\begin{array}{c} n\\ k \end{array}\right)}}},l_{t{\left(\prod_{1\leq i_{1}<\cdots <i_{k}\leq n}\left(1-{\left(\prod_{j=1}^{k}\left(1-{\left(\frac{\gamma_{i_{j}}}{t}\right)}^{w_{i_{j}}}\right)\right)}^{\frac{1}{k}}\right)\right)}^{\frac{1}{\left(\begin{array}{c} n\\ k \end{array}\right)}}}\right) \end{array}$$ (23)

Proof.

According to the operational rules of LNNs, we have

$$w_{i_{j}}e_{i_{j}}=\left(l_{t-t{\left(1-\frac{\alpha_{i_{j}}}{t}\right)}^{w_{i_{j}}}},l_{t{\left(\frac{\beta_{i_{j}}}{t}\right)}^{w_{i_{j}}}},l_{t{\left(\frac{\gamma_{i_{j}}}{t}\right)}^{w_{i_{j}}}}\right),$$

$$\prod_{j=1}^{k}w_{i_{j}}e_{i_{j}}=\left(l_{t\prod_{j=1}^{k}\left(1-{\left(1-\frac{\alpha_{i_{j}}}{t}\right)}^{w_{i_{j}}}\right)},l_{t-t\prod_{j=1}^{k}\left(1-{\left(\frac{\beta_{i_{j}}}{t}\right)}^{w_{i_{j}}}\right)},l_{t-t\prod_{j=1}^{k}\left(1-{\left(\frac{\gamma_{i_{j}}}{t}\right)}^{w_{i_{j}}}\right)}\right),$$

$$\mathrm{and}\;{\left(\prod_{j=1}^{k}w_{i_{j}}e_{i_{j}}\right)}^{\frac{1}{k}}=\left(l_{t{\left(\prod_{j=1}^{k}\left(1-{\left(1-\frac{\alpha_{i_{j}}}{t}\right)}^{w_{i_{j}}}\right)\right)}^{\frac{1}{k}}},l_{t-t{\left(\prod_{j=1}^{k}\left(1-{\left(\frac{\beta_{i_{j}}}{t}\right)}^{w_{i_{j}}}\right)\right)}^{\frac{1}{k}}},l_{t-t{\left(\prod_{j=1}^{k}\left(1-{\left(\frac{\gamma_{i_{j}}}{t}\right)}^{w_{i_{j}}}\right)\right)}^{\frac{1}{k}}}\right)$$

$$\mathrm{then}\;\sum_{1\leq i_{1}<\cdots <i_{k}\leq n}{\left(\prod_{j=1}^{k}e_{i_{j}}\right)}^{\frac{1}{k}}=\left(l_{t-t\prod_{1\leq i_{1}<\cdots <i_{k}\leq n}\left(1-{\left(\prod_{j=1}^{k}\left(1-{\left(1-\frac{\alpha_{i_{j}}}{t}\right)}^{w_{i_{j}}}\right)\right)}^{\frac{1}{k}}\right)},l_{t\prod_{1\leq i_{1}<\cdots <i_{k}\leq n}\left(1-{\left(\prod_{j=1}^{k}\left(1-{\left(\frac{\beta_{i_{j}}}{t}\right)}^{w_{i_{j}}}\right)\right)}^{\frac{1}{k}}\right)},l_{t\prod_{1\leq i_{1}<\cdots <i_{k}\leq n}\left(1-{\left(\prod_{j=1}^{k}\left(1-{\left(\frac{\gamma_{i_{j}}}{t}\right)}^{w_{i_{j}}}\right)\right)}^{\frac{1}{k}}\right)}\right),$$

$$\frac{1}{\left(\begin{array}{c} n\\ k \end{array}\right)}\sum_{1\leq i_{1}<\cdots <i_{k}\leq n}{\left(\prod_{j=1}^{k}e_{i_{j}}\right)}^{\frac{1}{k}}=\left(l_{t-t{\left(\prod_{1\leq i_{1}<\cdots <i_{k}\leq n}\left(1-{\left(\prod_{j=1}^{k}\left(1-{\left(1-\frac{\alpha_{i_{j}}}{t}\right)}^{w_{i_{j}}}\right)\right)}^{\frac{1}{k}}\right)\right)}^{\frac{1}{\left(\begin{array}{c} n\\ k \end{array}\right)}}},l_{t{\left(\prod_{1\leq i_{1}<\cdots <i_{k}\leq n}\left(1-{\left(\prod_{j=1}^{k}\left(1-{\left(\frac{\beta_{i_{j}}}{t}\right)}^{w_{i_{j}}}\right)\right)}^{\frac{1}{k}}\right)\right)}^{\frac{1}{\left(\begin{array}{c} n\\ k \end{array}\right)}}},l_{t{\left(\prod_{1\leq i_{1}<\cdots <i_{k}\leq n}\left(1-{\left(\prod_{j=1}^{k}\left(1-{\left(\frac{\gamma_{i_{j}}}{t}\right)}^{w_{i_{j}}}\right)\right)}^{\frac{1}{k}}\right)\right)}^{\frac{1}{\left(\begin{array}{c} n\\ k \end{array}\right)}}}\right)$$

Therefore,

$$WLNHM^{\left(k\right)}(e_{1},e_{2},\ldots .,e_{n})=\left(l_{t-t{\left(\prod_{1\leq i_{1}<\cdots <i_{k}\leq n}\left(1-{\left(\prod_{j=1}^{k}\left(1-{\left(1-\frac{\alpha_{i_{j}}}{t}\right)}^{w_{i_{j}}}\right)\right)}^{\frac{1}{k}}\right)\right)}^{\frac{1}{\left(\begin{array}{c} n\\ k \end{array}\right)}}},l_{t{\left(\prod_{1\leq i_{1}<\cdots <i_{k}\leq n}\left(1-{\left(\prod_{j=1}^{k}\left(1-{\left(\frac{\beta_{i_{j}}}{t}\right)}^{w_{i_{j}}}\right)\right)}^{\frac{1}{k}}\right)\right)}^{\frac{1}{\left(\begin{array}{c} n\\ k \end{array}\right)}}},l_{t{\left(\prod_{1\leq i_{1}<\cdots <i_{k}\leq n}\left(1-{\left(\prod_{j=1}^{k}\left(1-{\left(\frac{\gamma_{i_{j}}}{t}\right)}^{w_{i_{j}}}\right)\right)}^{\frac{1}{k}}\right)\right)}^{\frac{1}{\left(\begin{array}{c} n\\ k \end{array}\right)}}}\right)$$,

which Theorem 3 is proved.

Based on the operation rules of the LNNs, the WLNHM operator has also the same desirable properties described as follows:

**Property 1** (Commutativity). Let *e*_*i*_ (*i* = 1,2,⋯,*n*) be a collection of LNNs, and $\left(e_{1}^{\prime },e_{2}^{\prime },\ldots .,e_{n}^{\prime }\right)$ is any permutation of (*e*_1_,*e*_2_,….,*e*_*n*_), then

$$WLNHM^{\left(k\right)}(e_{1}^{\prime },e_{2}^{\prime },\ldots .,e_{n}^{\prime })=WLNHM^{\left(k\right)}(e_{1},e_{2},\ldots .,e_{n})$$ (24)

**Property 2** (Monotonicity). Let $e_{i}=\left(l_{\alpha_{i}},l_{\beta_{i}},l_{\gamma_{i}}\right)$, $f_{i}=\left(l_{p_{i}},l_{q_{i}},l_{r_{i}}\right)$ (*i* = 1,2,…,*n*) be two collections of LNNs, if *α*_*i*_ ≤ *p*_*i*_, *β*_*i*_ ≥ *q*_*i*_, *γ*_*i*_ ≥ *r*_*i*_ for all *i*, then

$$WLNHM^{\left(k\right)}(e_{1},e_{2},\ldots ,e_{n})\leq WLNHM^{\left(k\right)}(f_{1},f_{2},\ldots ,f_{n})$$ (25)

**Property 4 (**Boundedness). Suppose *e*^−^ = min(*e*_1_,*e*_2_,….,*e*_*n*_), *e*^+^ = max(*e*_1_,*e*_2_,….,*e*_*n*_) then

$$e^{-}\leq LNHM^{\left(k\right)}(e_{1},e_{2},\ldots .,e_{n})\leq e^{+}.$$ (26)

The proofs of the above theorems are similar with the corresponding theorems of LNHM so it’s omitted here.

## 4. Entropy of LNSs

Entropy is a useful tool to measure uncertainty in a set, including fuzzy set (FS), intuitionistic fuzzy set (IFS) and vague set etc. Here the LNS is characterized by handling uncertain information with truth-membership function, indeterminacy-membership function and falsity-membership function, respectively. As a consequence, it’s necessary to further define the entropy of LNS. Zadeh [38] first introduced the entropy of FS to measure fuzziness in 1965. Later De Luca-Termini [41] axiomatized the non-probabilistic entropy. Based on above studies, the entropy *E* of a fuzzy set *A* should satisfy the following axioms:

1. *E*(*A*) = 0 *iff A* ∈ 2^*X*^;
2. *E*(*A*) = 1 *iff μ*_*A*_ (*x*) = 0.5,∀*x* ∈ *X*;
3. *E*(*A*) ≤ *E*(*B*) *iff A* is less fuzzy than *B*, i.e. *if μ*_*A*_ (*x*) ≤ *μ*_*B*_ (*x*) ≤ 0.5,∀*x* ∈ *X* or *if μ*_*A*_ (*x*) ≥ *μ*_*B*_ (*x*) ≥ 0.5, ∀*x* ∈ *X*;
4. *E*(*A*^*C*^) = *E*(*A*).

About entropy measure, Kaufmann [42] proposed a distance based on the soft entropy. Kosko [43] introduced a new non-probabilistic entropy measure and investigated the degree of subsethood of one FS in another. Majumdar and Samanta [44] proposed several entropy measures for soft sets. Szmidt & Kacprzyk [45] studied the entropy of IFSs. Yager [46] put forward fuzziness measure in terms of distinction between the fuzzy set and its complement etc.

**Definition 12.** Assume that $A=\left\{\langle x_{i},l_{\alpha_{A}\left(x_{i}\right)},l_{\beta_{A}\left(x_{i}\right)},l_{\gamma_{A}\left(x_{i}\right)}\rangle |x_{i}\in X\right\}$ is a LNS, we define the entropy of LNS as a function *E*_*L*_: *L*(*X*)→[0,*t*], satisfying the following axiomatic requirements:

1. *E*_*L*_(*A*) = 0, if *A* is a crisp set;
2. $E_{L}\left(A\right)=1\;iff\;\frac{\alpha_{A}\left(x\right)}{t}=\frac{\beta_{A}\left(x\right)}{t}=\frac{\gamma_{A}\left(x\right)}{t}=0.5,\forall x\in X$;
3. *E*_*L*_(*A*) ≤ *E*_*L*_(*B*) *iff A* is less uncertain than *B*, i.e. $if\frac{\alpha_{A}\left(x\right)}{t}+\frac{\gamma_{A}\left(x\right)}{t}\geq \frac{\alpha_{B}\left(x\right)}{t}+\frac{\gamma_{B}\left(x\right)}{t}$ and $\left|\frac{\beta_{A}\left(x\right)}{t}-\frac{\beta_{A^{C}}\left(x\right)}{t}\right|\geq \left|\frac{\beta_{B}\left(x\right)}{t}-\frac{\beta_{B^{C}}\left(x\right)}{t}\right|$;
4. *E*_*L*_(*A*^*C*^) = *E*_*L*_(*A*).

As for the uncertain measure of LNS, we need to consider two factors, one is the partial truth-membership and partial false-membership and another is the indeterminacy factor. Based on these two factors we propose the entropy measure *E*_*L*_ of a LNS *A* as follows:

$$E_{L}\left(A\right)=1-\frac{1}{n}\sum_{x\in X}\left(\frac{\alpha_{A}\left(x\right)}{t}+\frac{\gamma_{A}\left(x\right)}{t}\right)\cdot \left|\frac{\beta_{A}\left(x\right)}{t}-\frac{\beta_{A^{C}}\left(x\right)}{t}\right|$$ (27)

Then we prove that (27) can meet the conditions of definition 12.

Proof:

1. For a crisp set *A* and there is no indeterminacy-membership for any LNN of *A*. Hence *E*_*L*_(*A*) = 0 holds.
2. If *A* be such that $\frac{\alpha_{A}\left(x\right)}{t}=\frac{\beta_{A}\left(x\right)}{t}=\frac{\gamma_{A}\left(x\right)}{t}=0.5,\forall x\in X$, then $\frac{\alpha_{A}\left(x\right)}{t}+\frac{\gamma_{A}\left(x\right)}{t}=1$ and $\frac{\beta_{A}\left(x\right)}{t}-\frac{\beta_{A^{C}}\left(x\right)}{t}=0.5-0.5=0,\forall x\in X\Rightarrow E_{L}\left(A\right)=1$.
3. *A* is less uncertain than *B*, we suppose $\frac{\alpha_{A}\left(x\right)}{t}+\frac{\gamma_{A}\left(x\right)}{t}\geq \frac{\alpha_{B}\left(x\right)}{t}+\frac{\gamma_{B}\left(x\right)}{t}$ and $\left|\frac{\beta_{A}\left(x\right)}{t}-\frac{\beta_{A^{C}}\left(x\right)}{t}\right|\geq \left|\frac{\beta_{B}\left(x\right)}{t}-\frac{\beta_{B^{C}}\left(x\right)}{t}\right|$. Based on the entropy value in Eq (27), we can obtain *E*_*L*_(*A*) ≤ *E*_*L*_(*B*).
4. $A^{C}=\left\{\langle x_{i},l_{\gamma_{A}\left(x_{i}\right)},l_{t-\beta_{A}\left(x_{i}\right)},l_{\alpha_{A}\left(x_{i}\right)}\rangle |x_{i}\in X\right\}$, $E_{L}\left(A^{C}\right)=1-\frac{1}{n}\sum_{x\in X}\left(\frac{\gamma_{A}\left(x\right)}{t}+\frac{\alpha_{A}\left(x\right)}{t}\right)\cdot \left|\frac{\beta_{A^{C}}\left(x\right)}{t}-\frac{\beta_{A}\left(x\right)}{t}\right|=E_{L}\left(A\right)$.

**Example 4.1** Let *X* be the universe set and *A* ={(*l*_3_,*l*_2_,*l*_1_),(*l*_6_,*l*_4_,*l*_2_),(*l*_5_,*l*_1_,*l*_3_),(*l*_5_,*l*_4_,*l*_3_)} be a LNS in *X*.

Then the entropy of *X* will be

$$\begin{array}{l} E_{L}\left(A\right)=1-\frac{1}{4}\left(\left(\frac{3}{6}+\frac{1}{6}\right)\cdot \left|\frac{2}{6}-\frac{6-2}{6}\right|+\left(\frac{6}{6}+\frac{2}{6}\right)\cdot \left|\frac{4}{6}-\frac{6-4}{6}\right|+\left(\frac{5}{6}+\frac{3}{6}\right)\cdot \left|\frac{1}{6}-\frac{6-1}{6}\right|+\left(\frac{5}{6}+\frac{3}{6}\right)\cdot \left|\frac{4}{6}-\frac{6-4}{6}\right|\right)\\ \;=1-0.5=0.5. \end{array}$$

## 5. The method for MAGDM based on the WLNHM operator

For a MAGDM with LNNs, let *A* = {*A*_1_,*A*_2_,….,*A*_*m*_} be a set of alternatives, *D* = {*D*_1_,*D*_2_,….,*D*_*t*_} be the set of DMs and *λ* = (*λ*_1_,*λ*_2_,….,*λ*_*t*_)^*T*^ be the weight vector of DMs *D*_*h*_(*h*=1,2,….,*t*), *λ*_*h*_ ∈ [0,1],*h* = 1,2,….,*t* and $\sum_{h=1}^{t}\lambda_{h}=1$. Let *C* = {*C*_1_,*C*_2_,….,*C*_*n*_}be the set of attributes and there are two kinds of attributes, i.e., the benefit attributes and the cost attributes. Here we assume the weight vector of the attributes is unknown. If the *h*th (*h* = 1,2,….,*t*) DM provides the evaluation of the alternative *A*_*i*_(*i* = 1,2,….,*m*) about the attribute *C*_*j*_(*j* = 1,2,….,*n*) based on the LTS, such as *s* = {*s*_0_ = *extremely poor*,*s*_1_ = *very poor*,*s*_2_ = *poor*,*s*_3_ = *slightly poor*,*s*_4_ = *medium*,*s*_5_ = *slightly better*,*s*_6_ = *good*, *s*_7_ = *very good*, *s*_8_ = *perfect*}, by the form of a LNN $x_{ij}^{h}=\left(l_{\alpha_{ij}}^{h},l_{\beta_{ij}}^{h},l_{\gamma_{ij}}^{h}\right)$ for $\alpha_{ij}^{h},\beta_{ij}^{h},\gamma_{ij}^{h}\in \left[0,t\right]$ (*h* = 1,2,….,*t*;*i* = 1,2,….,*m*;*j* = 1,2,….,*n*). Therefore, we can obtain the *h*th LNN decision matrix $X^{h}={\left[x_{ij}^{h}\right]}_{m\times n}$. Based on these information, the proposed MAGDM method can be presented as follows:

***Step 1*:** Standardize the decision-making information.

In general, if there exist cost attributes, then normalize each decision matrix $X^{h}={\left[x_{ij}^{h}\right]}_{m\times n}$ (*h* = 1,2,…,*t*) into the transformed decision matrix $R^{h}={\left[r_{ij}^{h}\right]}_{m\times n}$ (*h* = 1,2,…,*t*), where $x_{ij}^{h}=\left(l_{\alpha_{ij}}^{h},l_{\beta_{ij}}^{h},l_{\gamma_{ij}}^{h}\right)$ and

$$r_{ij}^{h}=\left(l_{p_{ij}}^{h},l_{q_{ij}}^{h},l_{r_{ij}}^{h}\right)=\{\begin{array}{c} \left(l_{\alpha_{ij}}^{h},l_{\beta_{ij}}^{h},l_{\gamma_{ij}}^{h}\right)\;for\;the\;benefit\;attribute\;C_{j}\\ \left(l_{t-\alpha_{ij}}^{h},l_{t-\beta_{ij}}^{h},l_{t-\gamma_{ij}}^{h}\right)\;for\;the\;\cos t\;attribute\;C_{j} \end{array}$$ (28)

where *h* = 1,2,….,*t*; *i* = 1,2,….,*m*; *j* = 1,2,….,*n*.

***Step 2*:** Aggregate all individual decision matrix *R*^*h*^ (*h* = 1,2,….,*t*) into collective one $R={\left[{\tilde{r}}_{ij}^{}\right]}_{m\times n}$.
  $${\tilde{r}}_{ij}=\left(l_{p_{ij}},l_{q_{ij}}^{},l_{r_{ij}}^{}\right)=WLNHM^{\left(k\right)}\left(r_{ij}^{1},r_{ij}^{2},\ldots ,r_{ij}^{t}\right)$$ (29)
  where ${\tilde{r}}_{ij}^{}$ is the collective evaluation value for alternative *A*_*i*_ with respect to attribute *C*_*j*_.
***Step 3*:** Calculate the weight of the attributes by utilizing the entropy of LNSs.

$$R_{j}=\left({\tilde{r}}_{1j},{\tilde{r}}_{2j},\ldots ,{\tilde{r}}_{mj}\right)$$

$$E_{L}\left(R_{j}\right)=1-\frac{1}{m}\sum_{x\in X}\left(\frac{\alpha_{R_{j}}\left(x\right)}{t}+\frac{\gamma_{R_{j}}\left(x\right)}{t}\right)\cdot \left|\frac{\beta_{R_{j}}\left(x\right)}{t}-\frac{\beta_{R_{j}^{C}}\left(x\right)}{t}\right|$$

$$\omega_{j}=E_{L}\left(R_{j}\right)/\sum_{j=1}^{n}E_{L}\left(R_{j}\right)$$ (30)

***Step 4*:** Calculate the comprehensive evaluation value of each alternative.
  $${\tilde{r}}_{i}=\left(l_{p_{i}},l_{q_{i}}^{},l_{r_{i}}^{}\right)=WLNHM^{\left(k\right)}\left({\tilde{r}}_{i1},{\tilde{r}}_{i2},\ldots ,{\tilde{r}}_{in}\right)$$ (31)
  where ${\tilde{r}}_{i}$ is the obtained comprehensive collective value of alternative *A*_*i*_, where *i* = 1,2,….,*m*.
***Step 5*:** Compute the score function $\varphi \left({\tilde{r}}_{i}\right)$ and the accuracy function $\sigma \left({\tilde{r}}_{i}\right)$ based on the Eqs (11) and (12).
***Step 6*:** Based on Definition 8, rank *A*_*i*_ (*i* = 1,2,….,*m*) in descending order. The larger the score function $\varphi \left({\tilde{r}}_{i}\right)$, the better the ranking order of alternative *A*_*i*_. If the score functions of the alternatives are the same, then the larger the accuracy function $\varphi \left({\tilde{r}}_{i}\right)$ of alternative *A*_*i*_, the better the ranking order of alternative *A*_*i*_ (*i* = 1,2,….,*m*).

## 6. Application examples

In the following, an illustrative example about the selection of investment alternatives from [22] is provided to show the advantages of the proposed method.

There are four companies as a set of alternatives *A* = {*A*_1_,*A*_2_,*A*_3_,*A*_4_}, where *A*_1_ is a car company; *A*_2_ is a food company; *A*_3_ is a car company and *A*_4_ is an arms company. And there are five attributes to be considered: (1) *C*_1_ is the geological risk analysis; (2) *C*_2_ is the production risk analysis; (3) *C*_3_ is the market risk analysis; (3) *C*_4_ is the management risk analysis; (3) *C*_5_ is the social environment analysis. A group of three DMs *D*_*h*_ (*h* = 1,2,3) are invited to evaluate the attribute values by the LTSs *s* = {*s*_0_ = *extremely poor*,*s*_1_ = *very poor*,*s*_2_ = *poor*,*s*_3_ = *slightly poor*,*s*_4_ = *medium*,*s*_5_ = *slightly better*,*s*_6_ = *good*, *s*_7_ = *very good*, *s*_8_ = *perfect*} and assume the weight vector of the three DMs is $\lambda ={\left(\frac{1}{3},\frac{1}{3},\frac{1}{3}\right)}^{T}$. By the above information, the company needs to consider the relation among attribute for parameters *k*. So suppose here *k* = 2. DMs *D*_*h*_ (*h* = 1,2,3) gives the evaluation value of the alternative *A*_*i*_(*i* = 1,2,3,4) on the attribute *C*_*j*_(*j* = 1,2,3) by LNN, and then three LNN decision matrices $X^{h}={\left[x_{ij}^{h}\right]}_{m\times n}$ are constructed and listed in Tables 1–3.

**Table 1:** Linguistic neutrosophic decision matrix *X*^1^ given by *D*_1_.

	*C*_1_	*C*_2_	*C*_3_	*C*_4_	*C*_5_
*A*_1_	(*l*_1_,*l*_2_,*l*_1_)	(*l*_2_,*l*_3_,*l*_2_)	(*l*_4_,*l*_4_,*l*_3_)	(*l*_1_,*l*_5_,*l*_1_)	(*l*_3_,*l*_3_,*l*_2_)
*A*_2_	(*l*_2_,*l*_6_,*l*_2_)	(*l*_3_,*l*_8_,*l*_2_)	(*l*_2_,*l*_4_,*l*_1_)	(*l*_3_,*l*_1_,*l*_2_)	(*l*_1_,*l*_2_,*l*_1_)
*A*_3_	(*l*_2_,*l*_3_,*l*_1_)	(*l*_3_,*l*_2_,*l*_3_)	(*l*_1_,*l*_4_,*l*_1_)	(*l*_3_,*l*_5_,*l*_1_)	(*l*_5_,*l*_2_,*l*_4_)
*A*_4_	(*l*_3_,*l*_1_,*l*_2_)	(*l*_1_,*l*_7_,*l*_1_)	(*l*_4_,*l*_6_,*l*_3_)	(*l*_2_,*l*_5_,*l*_1_)	(*l*_4_,*l*_6_,*l*_4_)

**Table 2:** Linguistic neutrosophic decision matrix *X*^2^ given by *D*_2_.

	*C*_1_	*C*_2_	*C*_3_	*C*_4_	*C*_5_
*A*_1_	(*l*_1_,*l*_6_,*l*_1_)	(*l*_4_,*l*_3_,*l*_4_)	(*l*_2_,*l*_6_,*l*_2_)	(*l*_3_,*l*_5_,*l*_2_)	(*l*_5_,*l*_2_,*l*_4_)
*A*_2_	(*l*_1_,*l*_4_,*l*_1_)	(*l*_3_,*l*_2_,*l*_1_)	(*l*_2_,*l*_3_,*l*_4_)	(*l*_4_,*l*_0_,*l*_5_)	(*l*_2_,*l*_6_,*l*_4_)
*A*_3_	(*l*_3_,*l*_5_,*l*_2_)	(*l*_2_,*l*_4_,*l*_3_)	(*l*_1_,*l*_6_,*l*_5_)	(*l*_3_,*l*_5_,*l*_3_)	(*l*_2_,*l*_6_,*l*_1_)
*A*_4_	(*l*_2_,*l*_7_,*l*_2_)	(*l*_4_,*l*_6_,*l*_1_)	(*l*_3_,*l*_7_,*l*_2_)	(*l*_4_,*l*_4_,*l*_2_)	(*l*_3_,*l*_8_,*l*_4_)

**Table 3:** Linguistic neutrosophic decision matrix *X*^3^ given by *D*_3_.

	*C*_1_	*C*_2_	*C*_3_	*C*_4_	*C*_5_
*A*_1_	(*l*_2_,*l*_4_,*l*_1_)	(*l*_3_,*l*_5_,*l*_2_)	(*l*_5_,*l*_1_,*l*_4_)	(*l*_2_,*l*_6_,*l*_1_)	(*l*_3_,*l*_3_,*l*_2_)
*A*_2_	(*l*_1_,*l*_2_,*l*_1_)	(*l*_2_,*l*_4_,*l*_2_)	(*l*_1_,*l*_5_,*l*_3_)	(*l*_4_,*l*_2_,*l*_0_)	(*l*_0_,*l*_5_,*l*_6_)
*A*_3_	(*l*_2_,*l*_3_,*l*_3_)	(*l*_1_,*l*_5_,*l*_2_)	(*l*_2_,*l*_4_,*l*_5_)	(*l*_0_,*l*_4_,*l*_6_)	(*l*_3_,*l*_2_,*l*_4_)
*A*_4_	(*l*_2_,*l*_3_,*l*_2_)	(*l*_4_,*l*_2_,*l*_1_)	(*l*_1_,*l*_4_,*l*_3_)	(*l*_3_,*l*_4_,*l*_5_)	(*l*_0_,*l*_4_,*l*_5_)

### 6.1 Procedure of decision making based on the WLNHM operator

***Case 1*:** If the information about the attribute weights is completely unknown, then we use the proposed method to handle the above problem which the decision-making steps as follows:
***Step 1*:** Standardize the decision-making information.

As all the measured attribute values are the cost type, then we need normalize evaluation values by Eq (28). The normalized decision-making matrix are shown in Tables 4–6.

**Table 4:** Normalized decision matrix *R*^1^.

	*C*_1_	*C*_2_	*C*_3_	*C*_4_	*C*_5_
*A*_1_	(*l*_7_,*l*_6_,*l*_7_)	(*l*_6_,*l*_5_,*l*_6_)	(*l*_4_,*l*_4_,*l*_5_)	(*l*_7_,*l*_3_,*l*_7_)	(*l*_5_,*l*_5_,*l*_6_)
*A*_2_	(*l*_6_,*l*_2_,*l*_6_)	(*l*_5_,*l*_0_,*l*_6_)	(*l*_6_,*l*_4_,*l*_7_)	(*l*_5_,*l*_7_,*l*_6_)	(*l*_7_,*l*_6_,*l*_7_)
*A*_3_	(*l*_6_,*l*_5_,*l*_7_)	(*l*_5_,*l*_6_,*l*_5_)	(*l*_7_,*l*_4_,*l*_7_)	(*l*_5_,*l*_3_,*l*_7_)	(*l*_3_,*l*_6_,*l*_4_)
*A*_4_	(*l*_5_,*l*_7_,*l*_6_)	(*l*_7_,*l*_1_,*l*_7_)	(*l*_4_,*l*_2_,*l*_5_)	(*l*_6_,*l*_3_,*l*_7_)	(*l*_4_,*l*_2_,*l*_4_)

**Table 5:** Normalized decision matrix *R*^2^.

	*C*_1_	*C*_2_	*C*_3_	*C*_4_	*C*_5_
*A*_1_	(*l*_7_,*l*_6_,*l*_7_)	(*l*_4_,*l*_5_,*l*_4_)	(*l*_6_,*l*_2_,*l*_6_)	(*l*_5_,*l*_3_,*l*_6_)	(*l*_3_,*l*_6_,*l*_4_)
*A*_2_	(*l*_7_,*l*_4_,*l*_7_)	(*l*_5_,*l*_6_,*l*_7_)	(*l*_6_,*l*_5_,*l*_4_)	(*l*_4_,*l*_8_,*l*_3_)	(*l*_6_,*l*_2_,*l*_4_)
*A*_3_	(*l*_5_,*l*_3_,*l*_6_)	(*l*_6_,*l*_4_,*l*_5_)	(*l*_7_,*l*_2_,*l*_3_)	(*l*_5_,*l*_3_,*l*_5_)	(*l*_6_,*l*_2_,*l*_7_)
*A*_4_	(*l*_6_,*l*_1_,*l*_6_)	(*l*_4_,*l*_2_,*l*_7_)	(*l*_5_,*l*_1_,*l*_6_)	(*l*_4_,*l*_4_,*l*_6_)	(*l*_5_,*l*_0_,*l*_4_)

**Table 6:** Normalized decision matrix *R*^3^.

	*C*_1_	*C*_2_	*C*_3_	*C*_4_	*C*_5_
*A*_1_	(*l*_6_,*l*_4_,*l*_7_)	(*l*_5_,*l*_3_,*l*_6_)	(*l*_3_,*l*_7_,*l*_4_)	(*l*_6_,*l*_2_,*l*_7_)	(*l*_5_,*l*_5_,*l*_6_)
*A*_2_	(*l*_7_,*l*_6_,*l*_7_)	(*l*_6_,*l*_4_,*l*_6_)	(*l*_7_,*l*_3_,*l*_5_)	(*l*_4_,*l*_6_,*l*_8_)	(*l*_8_,*l*_3_,*l*_2_)
*A*_3_	(*l*_6_,*l*_5_,*l*_5_)	(*l*_7_,*l*_3_,*l*_6_)	(*l*_6_,*l*_4_,*l*_3_)	(*l*_8_,*l*_4_,*l*_2_)	(*l*_5_,*l*_6_,*l*_4_)
*A*_4_	(*l*_6_,*l*_5_,*l*_6_)	(*l*_4_,*l*_6_,*l*_7_)	(*l*_7_,*l*_4_,*l*_5_)	(*l*_5_,*l*_4_,*l*_3_)	(*l*_8_,*l*_4_,*l*_3_)

***Step 2*:** Aggregate all individual decision matrix *R*^*h*^ (*h* = 1,2,….,*t*) into collective one $R={\left[{\tilde{r}}_{ij}^{}\right]}_{m\times n}$ by the *WLNHM* operator in (29), and the results are shown in Table 7.

**Table 7:** Integration decision matrix *R*.

	*C*_1_	*C*_2_	*C*_3_	*C*_4_	*C*_5_
*A*_1_	(*l*_3.636_,*l*_6.389_,*l*_7.652_)	(*l*_2.239_,*l*_6.537_,*l*_7.022_)	(*l*_1.825_,*l*_6.641_,*l*_6.863_)	(*l*_3.012_,*l*_5.541_,*l*_7.547_)	(*l*_1.823_,*l*_6.998_,*l*_7.022_)
*A*_2_	(*l*_3.636_,*l*_6.389_,*l*_7.547_)	(*l*_2.459_,*l*_5.520_,*l*_7.419_)	(*l*_3.286_,*l*_6.359_,*l*_7.066_)	(*l*_1.831_,*l*_7.828_,*l*_7.549_)	(*l*_4.777_,*l*_6.200_,*l*_6.641_)
*A*_3_	(*l*_2.703_,*l*_6.537_,*l*_7.311_)	(*l*_3.012_,*l*_6.559_,*l*_6.998_)	(*l*_3.636_,*l*_5.971_,*l*_6.637_)	(*l*_3.652_,*l*_5.975_,*l*_6.813_)	(*l*_2.025_,*l*_6.766_,*l*_6.931_)
*A*_4_	(*l*_2.703_,*l*_6.646_,*l*_7.268_)	(*l*_2.279_,*l*_5.720_,*l*_7.652_)	(*l*_2.509_,*l*_5.233_,*l*_6.998_)	(*l*_2.239_,*l*_6.169_,*l*_7.105_)	(*l*_3.355_,*l*_4.295_,*l*_6.169_)

***Step 3*:** Calculate the weight of attributes by Eqs (27) and (30), and get

$$\begin{array}{l} E_{L}\left(R_{1}\right)=0.1759,\;E_{L}\left(R_{2}\right)=0.3656,\;E_{L}\left(R_{3}\right)=0.3802,\;E_{L}\left(R_{4}\right)=0.2147,\;E_{L}\left(R_{5}\right)=0.4364\\ \omega_{1}=0.1118,\;\omega_{2}=0.2324,\;\omega_{3}=0.2418,\;\omega_{4}=0.1365,\;\omega_{5}=0.2775. \end{array}$$

***Step 4*:** Obtain the comprehensive evaluation value of each alternative by the *WLNHM* operator in (31), then we can get

$${\tilde{r}}_{1}=\left(l_{0.529},l_{7.685},l_{7.843}\right),\;{\tilde{r}}_{2}=\left(l_{0.753},l_{7.696},l_{7.850}\right),\;{\tilde{r}}_{3}=\left(l_{0.683},l_{7.660},l_{7.782}\right),\;{\tilde{r}}_{4}=\left(l_{0.590},l_{7.460},l_{7.816}\right).$$

***Step 5*:** Compute the score function $\varphi \left({\tilde{r}}_{i}\right)$ (*i* = 1,2,3,4) based on the Eq (11), and obtain

$$\varphi \left({\tilde{r}}_{1}\right)=0.0417,\;\varphi \left({\tilde{r}}_{2}\right)=0.0503,\;\varphi \left({\tilde{r}}_{3}\right)=0.0517,\;\varphi \left({\tilde{r}}_{4}\right)=0.0548.$$

***Step 6*:** Rank the alternatives

According to the above score functions $\varphi \left({\tilde{r}}_{i}\right)$ (*i* = 1,2,3,4), the ranking result is *A*_4_ ≻ *A*_3_ ≻ *A*_2_ ≻ *A*_1_.

So, the best alternative is *A*_4_.

***Case 2*:** If the information about the attribute weights is given by DMs, and the weight vector is *ω*_*j*_ = (0.17,0.29,0.11,0.3,0.13)^*T*^, then we handle the above problem with the proposed method in which *Step 3* is omitted.

As mentioned above, the score functions $\varphi \left({\tilde{r}}_{i}\right)$ (*i* = 1,2,3,4) of *Case 2* can be obtained as follow: $\varphi \left({\tilde{r}}_{1}\right)=0.0428$, $\varphi \left({\tilde{r}}_{2}\right)=0.0457$, $\varphi \left({\tilde{r}}_{3}\right)=0.0514$, $\varphi \left({\tilde{r}}_{4}\right)=0.0512$. Based on Definition 8, the ranking result is *A*_3_ ≻ *A*_4_ ≻ *A*_2_ ≻ *A*_1_, which is different from the order of *Case 1* due to the diverse weights of attribute values.

In typical MADM approaches, weights of attributes can reflect the relative importance in decision making process. However, the information about attribute weights is completely unknown or incompletely known because of time pressure, data loss, and the DMs’ limited domain knowledge about the problem. To get the optimal alternatives, we should determine the weight vector of attributes by some methods. The attribute weights in *Case 2* are to determine usually according to the preference or judgments of DMs while the *Case 1* adopts the entropy concept to confirm the weights of attribute values which can effectively balance the influence of subjective factors. Therefore, it’s more objective and reasonable to apply entropy of LNS to assign weight for each attribute in the decision-making process.

### 6.2 Discuss the influence of the parameter *k*

In order to discuss the influence of the parameter *k*, we can adopt different values of parameter *k* in our proposed method to rank the alternatives, and the results are listed in Table 8.

**Table 8:** Ranking results by utilizing the different *k*.

	$\varphi \left({\tilde{r}}_{1}\right)$	$\varphi \left({\tilde{r}}_{2}\right)$	$\varphi \left({\tilde{r}}_{3}\right)$	$\varphi \left({\tilde{r}}_{4}\right)$	Ranking
*k* = 1	0.0431	0.0590	0.0541	0.0618	*A*_4_ ≻ *A*_2_ ≻ *A*_3_ ≻ *A*_1_
*k* = 2	0.0417	0.0502	0.0517	0.0548	*A*_4_ ≻ *A*_3_ ≻ *A*_2_ ≻ *A*_1_
*k* = 3	0.0412	0.0471	0.0508	0.0526	*A*_4_ ≻ *A*_3_ ≻ *A*_2_ ≻ *A*_1_

As we can see from Table 8, the ranking orders are different with the parameter *k* changes in this example. When *k* = 2 and *k* = 3, the ranking results are same, i.e., *A*_4_ ≻ *A*_3_ ≻ *A*_2_ ≻ *A*_1_, whereas it produces a different ranking result “*A*_4_ ≻ *A*_2_ ≻ *A*_3_ ≻ *A*_1_” when *k* = 1. Obviously, when *k* = 1, the proposed method doesn’t consider the interrelationship among the attributes, and ranking result is different from the ones when *k* = 2 and *k* = 3, which mean the interrelationships between two attributes or among three attributes are considered. This verifies that the proposed method based on the WLNHM can provide more flexibility and adaptability in information aggregation and take full advantage of parameter change to solve MAGDM problems in which there are interrelationships between the attributes. Furthermore, it is noted that the score values of each alternative is reducing along with the parameter *k* increases, which reflect the risk preferences of the DMs in practical situations. In real-world decision-making situations, DMs can choose the appropriate value in accordance with their risk preferences. That is, it is more effective for DMs to select adaptive value of the parameter *k* according to their risk attitude. If the DM favors risk, he/she can take the parameter as small as possible; if the DM dislikes risk, he can take the parameter as large as possible. Therefore, the proposed method provides a general and flexible way to express the DMs’ preference and/or real requirements by utilizing the different parameter *k* in the decision process. Inspired by the idea of MSM operator proposed by Qin and Liu [31], we usually take *k* = [*n*/2] to solve problems, where symbol [] is a round function and *n* is the number of elements that need to be aggregated, which is not only intuitive and feasible, but comprehensive. In such case, the risk preference of DMs is neutral and the interrelationships of each argument can be fully taken into account.

### 6.3 Further compared with other methods

In the following, we make a comparison of the proposed method based on the *WLNHM* operator with the ones of Liang et al.’s method [22] based on the extended TOPSIS model, Fang and Ye’s method [13] based on the linguistic neutrosophic number weighted arithmetic averaging (*LNNWAA*) operator and Fan et al.’s method [21] based on the LNN normalized weighted Bonferroni mean (*LNNNWBM*) operator for dealing with the same problem adopted from [22]. In order to guarantee the rationality and scientificity of the contrasting results, these methods should be based the same weight vector of the attributes, so we used the weight vector *ω*_*j*_ = (0.08,0.20,0.15,0.27,0.30)^*T*^ which is given from [22]. In addition, in order to make the contrast even more remarkable, we set the different parameter value (*k* = 1,2,3) in *Step* 2 and *Step* 4 to aggregate evaluation information of DMs and attribute values of each alternative for proposed method in this paper, and the comparison results are listed in Table 9.

**Table 9:** A comparison of the ranking results from different methods.

Methods	Score values $\varphi \left({\tilde{r}}_{i}\right)$	Ranking
Liang et al.’s method [19] based on the extend TOPSIS model	No	*A*_4_ ≻ *A*_2_ ≻ *A*_3_ ≻ *A*_1_
Fang and Ye’s method [6] based on the *LNNWAA* operator	$\varphi \left({\tilde{r}}_{1}\right)=0.494$, $\varphi \left({\tilde{r}}_{2}\right)=0.790$, $\varphi \left({\tilde{r}}_{3}\right)=0.649$, $\varphi \left({\tilde{r}}_{4}\right)=0.792$	*A*_4_ ≻ *A*_2_ ≻ *A*_3_ ≻ *A*_1_
Fan et al.’s method [5] based on the *LNNNWBM* operator (*p* = *q* = 1)	$\varphi \left({\tilde{r}}_{1}\right)=0.459$, $\varphi \left({\tilde{r}}_{2}\right)=0.477$, $\varphi \left({\tilde{r}}_{3}\right)=0.521$, $\varphi \left({\tilde{r}}_{4}\right)=0.540$	*A*_4_ ≻ *A*_3_ ≻ *A*_2_ ≻ *A*_1_
the proposed method based on the *WLNHM* operator (*k* = 1)	$\varphi \left({\tilde{r}}_{1}\right)=0.077$, $\varphi \left({\tilde{r}}_{2}\right)=0.683$, $\varphi \left({\tilde{r}}_{3}\right)=0.380$, $\varphi \left({\tilde{r}}_{4}\right)=0.684$	*A*_4_ ≻ *A*_2_ ≻ *A*_3_ ≻ *A*_1_
the proposed method based on the *WLNHM* operator (*k* = 2)	$\varphi \left({\tilde{r}}_{1}\right)=0.041$, $\varphi \left({\tilde{r}}_{2}\right)=0.047$, $\varphi \left({\tilde{r}}_{3}\right)=0.051$, $\varphi \left({\tilde{r}}_{4}\right)=0.053$	*A*_4_ ≻ *A*_3_ ≻ *A*_2_ ≻ *A*_1_
the proposed method based on the *WLNHM* operator (*k* = 3)	$\varphi \left({\tilde{r}}_{1}\right)=0.447$, $\varphi \left({\tilde{r}}_{2}\right)=0.259$, $\varphi \left({\tilde{r}}_{3}\right)=0.461$, $\varphi \left({\tilde{r}}_{4}\right)=0.473$	*A*_4_ ≻ *A*_3_ ≻ *A*_1_ ≻ *A*_2_

From Table 9, we can see that the proposed method based on the *WLNHM* operator (*k* = 1), Liang et al.’s method [22] based on the extended TOPSIS model, and Fang and Ye’s method [13] based on the *LNNWAA* operator produce the same ranking result. These results can easily be explained that these methods don’t consider interrelationships among attributes. So they can verify the validity of the proposed method when *k* = 1. Similarly, the same ranking results can be obtained by the proposed method based on the *WLNHM* operator (*k* = 2) and Fan et al.’s method [21] based on the *LNNNWBM* operator (*p* = *q* = 1), and they are different from the ones when *k* = 1. These results can easily be explained that these methods consider interrelationships between two attributes. So these results can also verify the validity of proposed methods in this paper. In addition, when *k* = 3, the ranking results produced by the proposed method are different from above methods [13,21,22] and the results by the proposed method when *k* = 1 and *k* = 2. The reason is that the proposed method considered interrelationships among three attributes.

Based on the above analysis, we can get that the method proposed in this paper is more general and flexible than these methods [13,21,22] by adopting the *WLNHM* operator with parameter *k*.

In the following, we will compare and analyze some advantages of our proposed method with the three methods. From the above analysis, we can summarize characteristics of these methods shown in Table 10.

**Table 10:** Characteristic comparisons of different methods.

Methods	Whether aggregate indeterminate information	Whether consider interrelationship between two arguments	Whether consider interrelationship of multi arguments	Whether determinate weight vector of attributes more objective
Liang et al.’s hmetod [19]	No	No	No	Yes
Fang and Ye’s method [6]	Yes	No	No	No
Fan et al.’s method [5]	Yes	Yes	No	No
the proposed method	Yes	Yes	Yes	Yes

As shown in Table 10, we can find that:

(1) Compared with the method based on the extended TOPSIS model [22]

Liang et al.’s method [22] adopts the extended TOPSIS model which cannot aggregate evaluation information and does not consider interrelationships among attributes, while the proposed method base on the *WLNHM* operator which can easily integrate indeterminate information and reflect interrelationships among attributes. The proposed aggregation operator has more advantages than the extended TOPSIS model because they can integrate different attribute values of each alternative to comprehensive values and then rank the alternatives while the extended TOPSIS model can only rank them.

It is worth noting that these two methods adopt objective weight measure to determinate the weight vector of attributes. The proposed method utilizes fuzzy entropy to measure the weight of each attribute, while the method in [22] applies the maximum deviation model to determine the weight vector of attribute. The maximizing deviation method only focus on the divergence of alternatives, and the entropy measure used in the proposed method pays attention to synthesize the truth-membership, falsity-membership and indeterminacy-membership of alternatives for a certain attributes. In other words, the entropy weight method can better reflect the determinacy/indeterminacy of various attributes-if the entropy value for an attribute is bigger across alternatives, it can provide more useful information. Then, the attribute would be distributed a bigger weight; Otherwise, such an attribute would be assigned a small weight. So our proposed method is more general and more reasonable than Liang et al.’s method [22] in practical applications.

(2) Compared with the method based on the *LNNWAA* operator [13]

For aggregation operators, the method by Fang and Ye [13] used the *LNNWAA* operator which does not consider interrelationships among attributes, whereas our proposed method adopts the *WLNHM* operator which can capture the interrelationship among the multi-input attributes.

However, there are interrelationships among attributes in above example, i.e., the production risk analysis *C*_2_ and the market risk analysis *C*_3_, the management risk analysis *C*_4_ and the social environment analysis *C*_5_ etc., the method proposed in [13] cannot take into account interrelationships among attributes to handle MAGDM problem. It would seem the method in this paper can obtain more reasonable result in decision-making process. In a word, the arithmetic averaging operator is only the special cases of *LNHM* operator, the method proposed in this paper are more typical and general than that by Fang and Ye [6].

(3) Compared with the method based on the *LNNNWBM* operator [21]

In the view of aggregation function, both of these two methods consider the interrelationship of the attributes. The main advantage of the *WLNHM* operator proposed in this paper is that it can capture the interrelationship among the multi-input attributes, while Fan et al.’s method [21] only present the interrelationship between two attributes. It can be concluded that the method proposed in this paper is more general than that in Fan et al.’s method [21] by adopting the *WLNHM* operator with parameter *k*. In this case, DMs have the choice to take appropriate value of the parameter *k* based on their subjective preference. So the *WLNHM* operator introduced in this paper is helpful for the DMs to make effective measure under more complex decision-making environments. In addition, our method can greatly simplifies the process of calculation by using a simple formula only with one parameter *k*, whereas Fan et al.’s method [21] based on the *LNNNWBM* operator shows some complex operations through two parameters *p* and *q*.

Based on the comparisons and analysis above, the proposed method based on the *WLNHM* operator can overcome the shortcomings of the methods [13,21,22] in the situation when there are relationships among multi-attributes. Moreover, the proposed method based on the entropy weight measure can provide reasonable and objective weight of each attribute and reduce the impact of subjective weights determined by DMs.

In a word, we have verified the effectiveness of the proposed method and shown the advantages for solving MAGDM problem with indeterminate and inconsistent information.

## 7. Conclusion

In this paper, we propose the LNNHM, WLNNHM operators. Then we investigate some desirable properties and further discuss their special cases when the parameter takes different values. Further, we define the entropy of LNS and apply it to determinate weights. Based on the WLNNHM operator and entropy weight measure, we develop a novel MAGDM method with LNNs. We demonstrate the feasibility and advantages of proposed method by comparing with the existing methods [13,21,22]. In the future research, we shall further develop other methods with LNNs, such as TODIM and VIKOR of LNNs, and apply them to handle MADM or MAGDM problems, especially when we need to consider incomplete, indeterminate and inconsistent information in the problems. On the other hand, we can develop the potential applications of the proposed method to different domains.
